# A Meta-Analysis of the Global Prevalence of Temporomandibular Disorders

**DOI:** 10.3390/jcm13051365

**Published:** 2024-02-28

**Authors:** Grzegorz Zieliński, Beata Pająk-Zielińska, Michał Ginszt

**Affiliations:** 1 Department of Sports Medicine, Medical University of Lublin, 20-093 Lublin, Poland; 2 Interdisciplinary Scientific Group of Sports Medicine, Department of Sports Medicine, Medical University of Lublin, 20-093 Lublin, Poland; 3 Department of Rehabilitation and Physiotherapy, Medical University of Lublin, 20-093 Lublin, Poland; michal.ginszt@umlub.pl

**Keywords:** prevalence, temporomandibular disorders, TMDs, Asia, South America, Europe, meta-analysis, TMD, Europe, female

## Abstract

**Background**: This meta-analysis aims to evaluate the proportion of people with TMDs in different studies, considering factors such as geographical region, patient age, and sample size. **Methods**: The search yielded 6984 articles on the incidence of TMDs. Finally, 74 studies with 172,239 subjects and 35,259 with TMDs were selected for final analysis. Analyses were performed using the R statistical language. **Results**: The incidence of TMDs in the world population was 34%. The age group 18–60 years is the most exposed to TMDs. From the data presented, we observed that for each continent, the female group was 9% to 56% larger than the male group. The highest female-to-male ratio (F:M) was reported in South America (1.56), whereas the lowest F:M ratio was reported in Europe (1.09), suggesting an almost equal distribution of males and females. **Conclusions**: This suggests that geographical location may play a role in the results of the studies. The prevalence of TMDs was significantly higher in South America (47%) compared to Asia (33%) and Europe (29%). Larger epidemiological studies of TMDs in African and Australian populations are recommended. In conclusion, both visual and statistical assessments suggest that the results of our meta-analysis are robust and unlikely to be significantly affected by publication bias. This suggests that geographical location may play a role in the prevalence of TMDs.

## 1. Introduction

Temporomandibular disorders (TMDs) is the term used to describe pain and dysfunction in the chewing muscles and temporomandibular joint (TMJ). TMDs are the most common oral and facial pain condition [1]. In addition, TMD signs and symptoms include a restricted range of motion and TMJ noises [2]. However, chronic pain is the main reason why patients with TMDs seek treatment [1]. Patients with TMDs are more likely to experience tinnitus [3] and sleep quality problems [4], and there are bidirectional associations between TMDs and depressive and anxiety disorders [5]. It has been estimated that treating TMDs costs $4 billion worldwide [6]. The most common treatments for TMDs include physiotherapy, occlusal splint therapy, and pharmacological treatment [7].

The etiology of TMDs is multifactorial. The main factors include biomechanical, neuromuscular, biopsychosocial, and biological factors: occlusal overload and parafunctions (e.g., bruxism) belong to the first group, biopsychosocial factors causing TMDs are stress, anxiety, or depression, and biological factors include, for example, increased levels of estrogen hormones [8].

For the diagnosis of TMDs, current studies use the Diagnostic Criteria for TMD (DC/TMD) developed in 2014 [9], the Diagnostic Criteria for Temporomandibular Disorders (RDC/TMD) developed in 1992 [10], the Fonseca Anamnestic Index (FAI) developed in 1994 [11], and the Short-Form Fonseca Anamnestic Index (SFAI) developed in 2018 [12], among others, including the Helkimo Indexes developed in 1974 [13], the Krogh-Poulsen criteria, the TMJ Scale, the Craniomandibular Index, and the Criteria of the American Academy of Orofacial Pain [14]. A large proportion of current epidemiological studies are based on the adaptation of any of the above protocols for example [15,16,17,18]. The examination procedure and diagnosis with the best statistical properties are currently DC/TMD and RDC/TMD [10,15]. Some of the questionnaires have similar accuracy. For example, the FAI and the SFAI have shown higher accuracy than the DC/TMD [19,20]. Compared with the RDC, the FAI showed high sensitivity and low specificity [21].

In the last five years, there has been one meta-analysis of the prevalence of TMDs in the global population [22], one meta-analysis of the prevalence of TMDs in children [23], and two meta-analyses of the prevalence in the Brazilian [24] and Chinese [25] populations. The meta-analyses of the global and Brazilian populations were based on the prevalence of TMDs based on RDC/TMD and DC/TMD [22,23,24]. As shown above, FAI and SFAI can effectively screen for TMDs [19,20,21]. Therefore, we believe that the exclusion of studies based on the exclusion of screening methods other than RDC/TMD or DC/TMD may have significantly influenced the results of these meta-analyses [22,23,24].

Due to the observed use of different tools and methods to study the prevalence of TMDs, a Total Quality Score (TQS) developed by Giannakopoulos et al. was used to assess the quality of prevalence studies [14]. This tool can be further used to assess the quality of scientific articles on prevalence studies. The quality assessment of prevalence studies consists of three main criteria: sampling, measurement, and analysis. Each criterion is addressed. Based on the information obtained from each article, the points assigned to each main criterion are summarised as a Total Quality Score (TQS). Finally, according to the TQS, the quality of the included articles was divided into four levels: 0–4 (poor); 5–9 (fair); 10–14 (good); and 15–19 (outstanding) [14]. According to Giannakopoulos et al., the TQS for ICC_(2,1)_ ranged from 0.94 to 1.00, indicating almost perfect agreement between researchers [14]. The abovementioned tool has been used in meta-analyses [25,26,27].

Given the above information, it was decided to conduct the present meta-analysis considering the TQS score and publications with good and outstanding results. The present meta-analysis aims to evaluate the proportion of individuals with TMDs across various studies, considering geographical region, patient age, and sample size. To the best of our knowledge, there has been no meta-analysis on the prevalence of TMDs according to geographical region.

## 2. Materials and Methods

We conducted a systematic search and review of TMD prevalence based on articles conducted from 1 January 1994 to 1 December 2022. The starting date was chosen because it was two years after introducing the RDC/TMD questionnaire [10] (which could have allowed for standardised research on TMDs). Additionally, it is the date of the introduction of the second standardised FAI protocol [11]. This systematic review is following the PRISMA guidelines, but was not registered.

We searched PubMed (National Library of Medicine) [28,29,30,31] from 9 January to 11 June 2023 for publications using MeSH (Medical Subject Heading) terms: temporomandibular disorders AND prevalence, temporomandibular disorders AND epidemiology, temporomandibular disorders AND population. Based on the work of Valesan et al., no restrictions on age, gender, or language of publication were applied. Both painful and non-painful TMDs were accepted [22].

The review was carried out by two independent reviewers (G.Z. and B.P.-Z.), who first evaluated the titles of the papers, then the abstracts, and finally the full papers. Any disputes that arose were evaluated by M.G. A summary of the PICO [32,33] standards (population, intervention, comparison, outcome), including inclusion and exclusion criteria, is found in Table 1.

The search yielded 6984 articles on the incidence of TMDs. Each publication’s titles and abstract were reviewed, and articles that were population-based studies were included. Studies were excluded if they were case studies or involved animal studies. In this step, 78 studies were evaluated according to TQS. The study rated the good and outstanding groups based on the TQS assessed (Supplementary Material S1).

**Figure 1:**
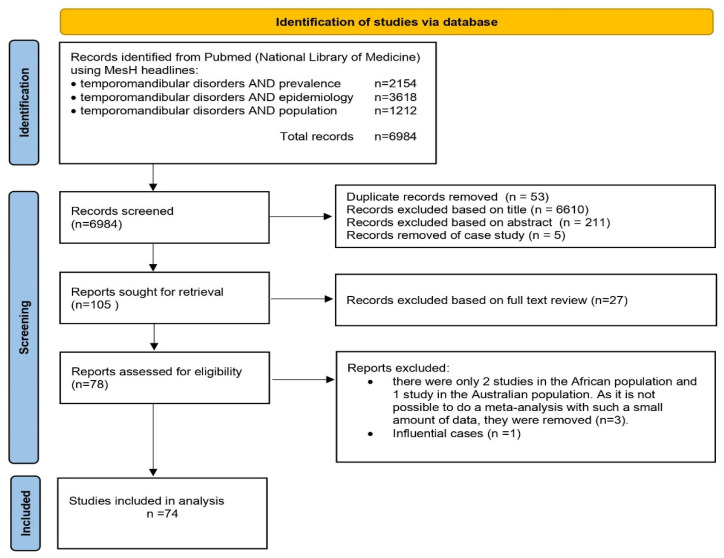
PRISMA flow diagram.

During the penultimate step of the evaluation of the 78 studies, it was noted that only 2 studies were from the African population [34,35], and 1 study was from the Australian population [36]. Because single studies for Australia and Africa were found, conducting a meta-analysis for these continents was impossible. One influential case [37] was identified during data pre-processing, and study [38] was removed from further analysis (Supplementary Material S5). Finally, 74 studies (Figure 1, Supplementary Material S2) were included, which analysed 80 populations (studies that analysed more than one population: Khan et al. analysis of TMDs incidence in Europe, North America, and South America [16], Wu et al. European and Asian populations [39], Hongxing et al. European and Asian populations [40], and De Stefano et al. European and South American populations [41]). In order to determine the age of onset of TMDs in each continent, an age classification was made as follows: “up to 18 yrs. (years)”, “18–60 yrs.” and “60+ yrs.” [42]. In addition, a study by Yekkalam and Wänman on the Swedish population was divided into two age groups (“18–60 yrs.” and “60+ yrs.”) for meta-analysis [43].

### 2.1. Characteristics of the Sample

Based on an earlier meta-analysis, we set the minimum sample size at 11,500 subjects [22]. The meta-analysis aimed to pool results from k = 80 studies, comprising a total of 172,239 subjects and 35,259 with TMDs as a dichotomous dependent variable. The TMD metric represented the number of individuals with TMD occurrence relative to the total number of individuals in each study (a proportion meta-analysis without a control group).

The studies are classified based on several factors:

- Geographical region: studies were categorized by the continent where the research was conducted, including Asia, Europe, South America, and North America;
- Age group: patient age is categorized into three distinct groups—younger patients (up to 18 yrs.), young and middle-aged patients (18–60 yrs.), older patients (over 60 yrs.);
- Sample size: includes the overall size of the sample and the gender distribution within the sample (number of males and females).

### 2.2. Statistical Analysis

#### 2.2.1. Significance Level

The significance level of the statistical tests in this analysis was set at α = 0.05.

#### 2.2.2. Effect Size Estimation

Each study’s proportion of TMDs was computed as the ratio of MDR occurrences to the sample size. Given the non-normal distribution of these proportions, they were transformed into log ratios or log odds. The corresponding sample variance was then estimated for each study. A random effects model was applied to calculate the logit proportion across all studies, with heterogeneity (τ^2^) evaluated using the DerSimonian–Laird estimator [44,45], and its confidence interval was determined using the Jackson method [46].

#### 2.2.3. Identifying Outliers and Influential Cases

Outliers were detected by ‘studentizing’ the residuals from the random effects model. Any absolute residual with a z-score exceeding 2.0 was classified as an outlier in this scenario.

To assess the influence of these outliers, we employed visual diagnostic techniques, specifically the leave-one-out method [37]. This process entailed examining each case in isolation, using metrics such as the externally standardized residual, DFFITS value, Cook’s distance, and covariance ratio. Additionally, we evaluated the leave-one-out amount of (residual) heterogeneity, the leave-one-out test statistic for (residual) heterogeneity, and DFBETAS values.

#### 2.2.4. Subgroup Analysis

For a more granular understanding of heterogeneity, we conducted a subgroup analysis. Studies were stratified based on one or more factors under investigation. The procedure involved computing aggregate proportions and their corresponding 95% confidence intervals for each subgroup using a random effects model. Both within-study and between-study variances were estimated in this process. The inverse variance method [47] was applied to establish the weight of individual studies.

Heterogeneity within subgroups was assessed using the I^2^ statistic, the Q statistic, τ^2^, and τ. The I^2^ statistic and Q statistic were used to evaluate the percentage of total variation across studies due to heterogeneity rather than chance. The τ^2^ and τ values, representing the estimated between-study variance and standard deviation, respectively, provided further measures of heterogeneity within each subgroup. The Q statistic was also utilized to test for significant differences in effect size across the defined subgroups.

The results of the subgroup analysis were also visualized in the form of a forest plot.

#### 2.2.5. Publication Bias

Publication bias was evaluated using a comprehensive, multi-step approach to provide a thorough and robust assessment. First, we constructed a funnel plot based on a random effects model. This graphical representation provided an intuitive way to visually assess publication bias.

Next, we used the ‘trim and fill’ method, an iterative procedure developed by Duval and Tweedie [48,49], to adjust for potential publication bias in our meta-analysis. This method seeks to identify and ‘fill’ any gaps in the funnel plot, which represent potentially missing studies due to publication bias. The imputed studies were used to recalculate a corrected pooled-effect size, providing an estimate of the effect size that might have been observed in the absence of publication bias.

Following the graphical assessment, we statistically evaluated funnel plot asymmetry using Egger’s regression test [50]. This test uses a linear regression model with the study effect sizes as the response variable and their standard errors as the predictor.

Finally, we performed the rank correlation test established by Begg and Mazumdar [51] to investigate whether there was a correlation between the effect sizes (or outcomes) and their corresponding sample variances.

#### 2.2.6. Statistical Environment

Analyses were conducted using the R statistical language (version 4.1.1; R Core Team, 2021) on Windows 10 Pro 64 bit (build 19045), using the packages meta (version 6.0.0; [52]), weightr (version 2.0.2; [53]), report (version 0.5.7; [54]), metasens (version 1.5.0; [55]), metafor (version 3.8.1; [56]), ggplot2 (version 3.4.0; [57]), readxl (version 1.3.1; [58]), dplyr (version 1.1.2; [59]), dmetar (version 0.0.9000; [60]), and scales (version 1.2.1; [61]).

## 3. Results

The heterogeneity analysis of the subgroups reveals some patterns. Asia and South America display moderate between-study variance, as indicated by their τ^2^ values. On the other hand, Europe and North America demonstrate considerably higher between-study variance. The Q values for Europe and Asia were particularly high, underscoring the fact that the observed effect sizes within these continents deviate significantly from what could be attributed to sampling error alone. This suggested a substantial degree of variation in the studies within these regions. Moreover, the I^2^ values for all continents were strikingly high, nearing 100%. This indicated that almost all the variability in the effect sizes across the studies within each continent stemmed from true heterogeneity, i.e., real differences in effect size, as opposed to chance variations.

**Figure 2:**
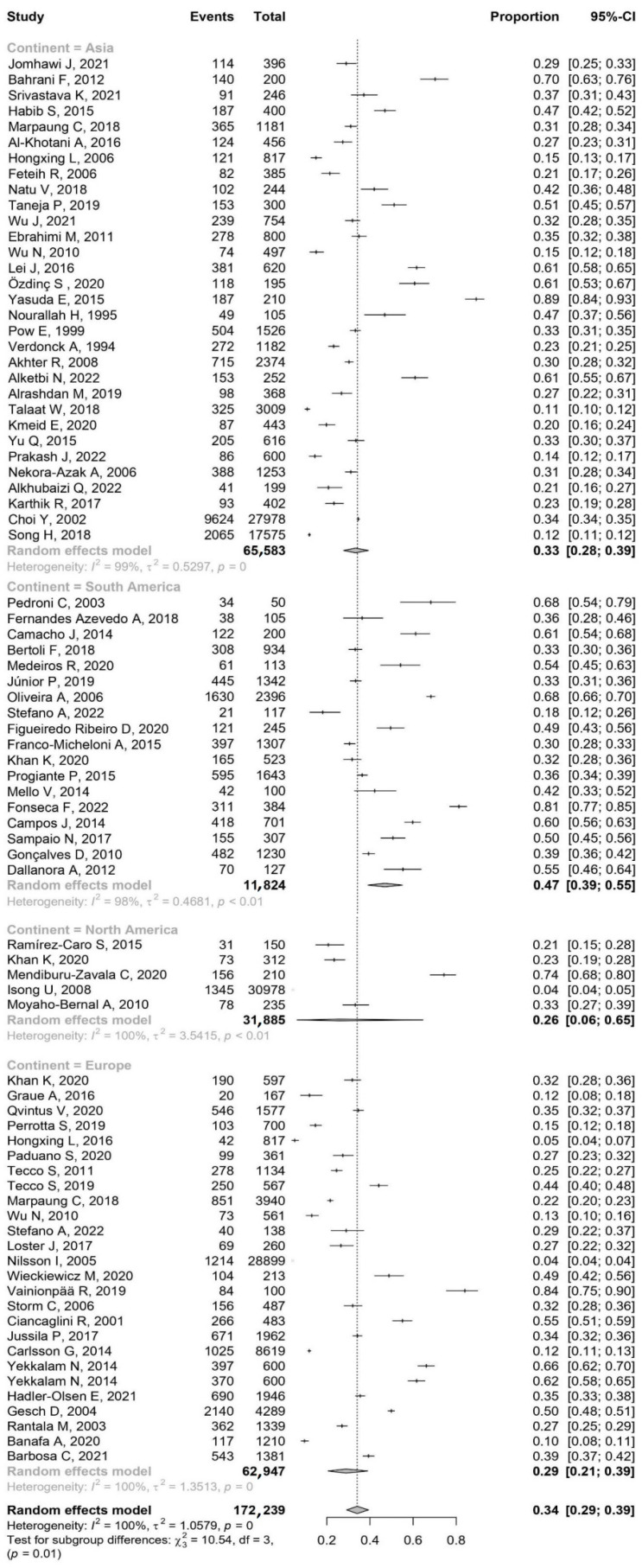
The results by forest plot prevalence of TMDs by continent (Supplementary Materials S2 and S3).

A test for subgroup differences, Q(3) = 10.54, yields a *p* = 0.015, denoting statistically significant differences between the continents. This suggests that geographical location might play a role in the outcome of the studies. Upon further analysis of confidence intervals, it becomes evident that the prevalence of TMDs was significantly higher in South America compared to Asia and Europe. However, no significant differences were observed among the other groups (Figure 2).

**Figure 3:**
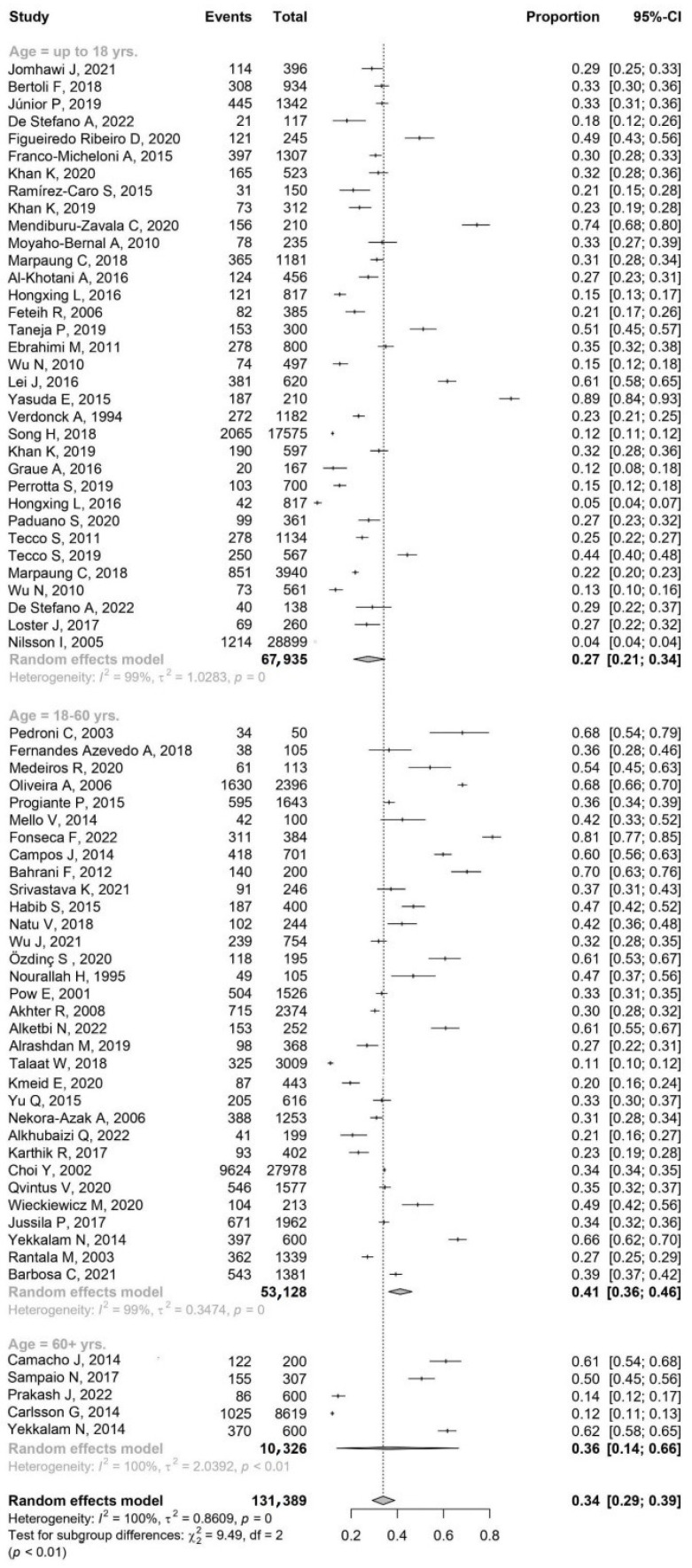
The results by forest plot prevalence of TMDs by age (Supplementary Materials S2 and S3).

The data (Figure 3) suggested that the age group of 18–60 years has the highest proportion of TMDs, followed by the 60+ years group, and then the group up to 18 years. However, due to the large confidence interval in the 60+ years group, it was unclear if the true proportion might actually be higher or lower than the other groups.

The difference in the proportion of TMDs cases among the age groups could be influenced by the high heterogeneity within each group. The highest heterogeneity is observed in the 60+ years group, which could explain the wide confidence interval and the uncertainty around the proportion of TMDs in this age group. While the number of studies might influence the heterogeneity to some extent, it did not appear to be the primary determinant. The relatively lower heterogeneity in the 18–60 years group could contribute to a more consistent and higher proportion of TMDs observed in this group across different studies. For the group up to 18 years, the high heterogeneity might reflect the diversity of factors influencing TMDs in this young population, thus impacting the observed proportion.

**Figure 4:**
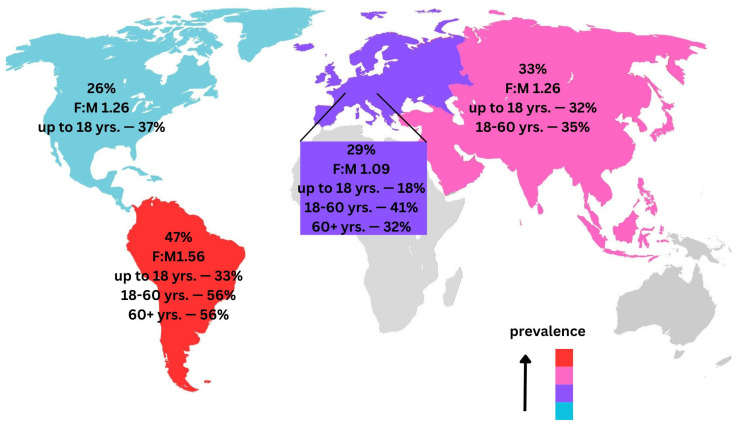
Prevalence of TMDs by continent, age, and female-to-male ratio (F:M).

A test for subgroup differences, represented as Q(2) = 9.49, *p* = 0.009, signified a statistically significant variation among different age brackets. This finding implied a potential influence of age on the study outcomes. On delving deeper into the confidence intervals, it becomes clear that TMDs were notably more prevalent in patients aged between 18–60 years compared to those aged 18 years and below. This marked difference underscores the potential influence of age on TMDs prevalence. However, the analysis does not indicate any significant differences when comparing other age groups (Figure 3 and Figure 4).

### 3.1. The Prevalence of TMDs by Continent and Age

#### 3.1.1. Asia

The test for subgroup differences using a random effects model yielded a Q(2) = 2.79 between groups. The *p*-value for the between-group differences was 0.2483, indicating that the differences in TMDs diagnosis proportions across the age groups were not statistically significant.

The age group 18–60 showed a proportion of TMDs diagnoses close to and slightly higher than the overall mean sample proportion. The age group up to 18 also demonstrated a similar trend but with a slightly lower proportion. In contrast, the age group of 60 and above exhibited a significantly lower proportion of TMDs diagnoses than the mean sample proportion (Figure 4 and Supplementary Material S4).

#### 3.1.2. South America

The test for subgroup differences using a random effects model yielded a Q(2) = 22.89, *p* < 0.001. This indicated that the differences in TMDs diagnosis proportions across the age groups were statistically significant. The proportions of TMDs diagnoses in the 18–60 yrs. and 60+ age yrs. groups were significantly higher than in the age group up to 18 years. The 18–60 age group also showed the highest variability across studies. These findings suggest that age may be an important factor influencing the prevalence of TMDs diagnoses, with more variability observed in the middle-aged group (Figure 4 and Supplementary Material S4).

#### 3.1.3. North America

The proportion of TMDs diagnoses in the “Up to 18 years” subgroup was slightly higher than the mean sample proportion. However, the confidence interval for this age group was wider than that of the mean sample proportion, indicating a high degree of uncertainty around the estimate. This is reflected in the high τ^2^ value, which suggested considerable heterogeneity in the results of the individual studies within this age group (Figure 4 and Supplementary Material S4).

#### 3.1.4. Europe

Comparing these to the mean sample proportion (0.34 with a 95% CI [0.29, 0.39]), we note: that the “up to 18” age group had a lower TMDs diagnoses proportion than the mean; the “18–60” age group had a higher TMDs diagnoses proportion than the mean; the “60+” age group, despite its lower TMDs diagnoses proportion, exhibited substantial heterogeneity.

The test for subgroup differences using a random effects model yielded a Q(2) = 8.31 with a *p*-value of 0.016. This indicated that the differences in TMDs diagnosis proportions across the age groups were statistically significant. Based on the confidence intervals, there was a significantly lower proportion of TMDs in the up to 18 years compared with 18–60 yrs. (Figure 4 and Supplementary Material S4).

#### 3.1.5. Prevalence of TMDs Female-to-Male Ratio (F:M)

From the data presented, we observed that for each continent, the size of the female group was larger than the male group by an average of 9% to 56%. The highest F:M was reported in South America (1.56). Conversely, Europe reported the lowest F:M (1.09), suggesting a near-equal distribution of males and females. However, the Kruskal–Wallis test, a non-parametric method used to compare two or more independent samples of equal or different sample sizes, yielded a *p*-value of 0.286.

An extended description of the results is available in the Supplementary Material S3.

## 4. Discussion

The present meta-analysis aims to evaluate the proportion of individuals with TMDs across various studies, considering geographical region, patient age, and sample size. The incidence of TMDs in the world population was 34% (Asia—33%, South America—47%, North America—26%, Europe—29%). The sheer number of TMDs incidences in the population underlines the importance of this disease entity. It represents an important economic and social problem. Further research into its diagnosis and therapy is important.

As for the age distribution, in the group “up to 18 yrs.”—27%, “18–60 yrs.”—41% and in the group “60+ yrs.”—36%. According to the meta-analysis by Valesan et al., it was approximately 31% for adults/elderly and 11% for children/adolescents [22]. Our meta-analysis’s results were similar to Valesan et al.’s observations. According to the meta-analysis by Minervini et al., the prevalence of TMDs in children and adolescents (subjects evaluated aged 8–19 years) varies between 20% and 60% [23]. In our area, up to the age of 18 years, the average prevalence of TMDs was 27% (Asia—32%, South America—33%, North America—37%, Europe 18%). The average prevalence of TMDs and the results for Asia, South America, and North America fall within the range defined by Minervini et al. [23]. The meta-analysis by Melo et al. aimed to assess the prevalence of TMDs in the Brazilian population. They found that the prevalence of TMDs was 33.6% [24]. Our analysis was 47%, but other South American countries were included. The meta-analysis by Xie et al. showed that the prevalence of TMDs in Chinese students was approximately 29.1% [25]. The age group of students is the group over 18 years old. In our study, it was the 18–60 yrs. group and their results for Asia were 35%. Considering that the 18–60 yrs. group includes not only students but also working people, this result can be considered similar to the work of Xie et al. The results we obtained, which are consistent with the above meta-analyses, may suggest the effectiveness of TQS in trial evaluation.

A result that is particularly noteworthy is the prevalence of TMDs in South America, at 47% (“up to 18 yrs.”—33%, “18–60 yrs.”—56%, “60+ yrs.”—56%). This result raises further research questions about the relationship of TMDs to either geographic region, cultural or anthropometric features. To date, the evidence has been unclear that cultural, geographic, socioeconomic, and gender factors contribute to the impact of altered masticatory function [62]. Our results demonstrate that the geographic factor is important in the occurrence of TMDs. We observed a higher prevalence of TMDs in women. This confirms previous scientific studies and the results of meta-analyses [22,23,24,25].

The higher prevalence of TMDs in South America may be related to sociodemographic [63], and psychosocial [8,64] factors. The prevalence of TMDs has also been associated with genetic factors [65]. However, further research is needed to find a factor directly related to the observed higher prevalence of TMDs in South America.

Warren and Fried suggest that the sex and age distribution of TMDs suggests a possible link between its pathogenesis and the female hormonal axis [66]. A study by Jedynak et al. suggests that extended female endocrine dysfunction may be associated with TMDs [67]. The observations in this meta-analysis associated with a higher prevalence of TMDs in women may be related to endocrine function. However, further research is needed.

A limitation of the present meta-analysis is that there is no breakdown of the types of TMDs (including joint pain, disc displacement, and osteoarthritis). This is because not every research method and study itself defines this division. This is the problem of epidemiological research on TMDs. This problem may be due to the different research tools, which, despite the high results obtained in the TQS, should be standardized in future studies with one specific tool. The second limitation is that our meta-analyses do not distinguish between painful and non-painful forms of the dysfunction. This means that we cannot speculate about the need and method for treatment. The third limitation is the lack of analysis of the incidence of TMDs in Africa and Australia. The lack of research in this area makes conducting a broader data analysis and objective conclusions impossible. Therefore, future studies should focus on the incidence in these two continents.

## 5. Conclusions

- This study suggests that geographical location may play a role in the epidemiology of TMDs. The prevalence of TMDs was significantly higher in South America (47%) compared to Asia (33%) and Europe (29%).
- This study suggests a higher incidence of TMDs in females compared to males in these age groups compared to the 18–60 age group.
- From the data presented, the female group was, on average, 9% to 56% larger than the male group in each continent. The highest female-to-male ratio was reported in South America (1.56), whereas the lowest F:M was reported in Europe (1.09), suggesting an almost equal distribution of males and females.
- Larger epidemiological studies of TMDs in African and Australian populations are recommended.
- In conclusion, both visual and statistical assessments suggest that the results of our meta-analysis are robust and unlikely to be significantly affected by publication bias.

## Figures and Tables

**Table 1:** PICO Summary of Inclusion and Exclusion Criteria.

	Inclusion	Exclusion
Patient		
	Adult and pediatric population	
Intervention		
	Screening for TMDs	
Outcome		
	Information in the study on: epidemiology, prevalence, TMD population. Research rated as good and outstanding by TQS.	Research rated as poor and fair by TQS.
Comparison		
	TMDs vs. Subject health	
Study Design		
	Studies to assess occurrence of TMDs in populations. The criterion for continents is at least 2 studies with a total of more than 500 participants.	Narrative Review Systematic Articles Meta-analysis Opinions Case reports or series patients Animal or biomechanical studies Publications in a language other than English Post-conference abstracts

TMDs—Temporomandibular disorders; TQS—Total Quality Score.

## Data Availability

Not applicable.

## Supplementary Materials

The following supporting information can be downloaded at: https://www.mdpi.com/article/10.3390/jcm13051365/s1, Supplementary Material S1: Total Quality Score result of the analysed papers; Supplementary Material S2: List of 74 studies included the meta-analysis; Supplementary Material S3: An extended description of the materials and methods, results; Supplementary Material S4: The meta-analysis results by forest plot; Supplementary Material S5: Rejected studies from the meta-analysis.
