# Supplementary material for: A Meta-Analysis of the Global Prevalence of Temporomandibular Disorders
Source: J Clin Med. 2024 Feb 28;13(5):1365. doi: 10.3390/jcm13051365 (PMC10931584; doi:10.3390/jcm13051365)
Supplement: Supplementary file 1 [file jcm-13-01365-s001.zip › Supplementary Material S1.pdf]

Total Quality Score of the analysed papers.

No.	First Author	Year	ID	Ethic Commission Approval	Recruitment Procedure	Response Rate Enough?	Is the Rate Taken Into Account?	Power	Do the Respondents Match the Target?	Standardized Data-Collection Methods	Reliable Survey Instruments?	Valid Survey Instruments?	Were Special Features Accounted For?	Satisfactory Confidence Intervals?	Total Quality Score	Quality Subgroups
1	Akhter, R.	2008	PMID: 18351034	0	2	1	0	2	4	2	0	0	1	1	13	(good)
2	Alketbi, N.	2022	10.12688/f1000research.109696.2	1	2	NA	0	2	3	2	2	2	0	0	14	(good)
3	Al-Khotani, A.	2016	10.1186/s10194-016-0642-9	1	2	NA	0	2	3	2	2	2	1	0	15	(outstanding)
4	Alkhubaizi, Q.	2022	10.1155/2022/3186069	1	2	0	0	2	3	2	1	0	0	0	11	(good)
5	Alrashdan, M.	2019	10.1111/jicd.12390	1	2	NA	0	2	4	2	2	2	1	1	17	(outstanding)
6	Bahrani, F.	2012	10.5005/jp-journals-10024-1116	0	2	NA	0	2	3	2	0	1	0	0	10	(good)
7	Banafa, A.	2020	10.1080/00016357.2019.1650955	0	2	1	0	2	4	2	0	0	0	0	11	(good)
8	Barbosa, C.	2021	10.1111/joor.13226	0	2	1	0	2	3	2	0	1	1	1	13	(good)
9	Bertoli, F.	2018	10.1371/journal.pone.0192254	1	2	NA	0	2	4	2	2	2	1	1	17	(outstanding)

10	Camacho, D.	2014	/10.1590/S2317-17822014000100011	1	2	NA	0	2	3	2	0	1	0	0	11	(good)
11	Campos, J.	2014	10.11607/ofph.1194	1	2	1	0	2	4	2	0	0	0	0	12	(good)
12	Carlsson, G.	2014	00016357.2014.898787	1	2	0	0	2	4	2	0	1	0	0	12	(good)
13	Choi, Y.-S	2002	10.1053/joms.2002.33249	0	2	NA	0	2	4	2	0	1	0	1	12	(good)
14	Ciancaglini, R.	2001	10.1016/S0300-5712(00)00042-7	0	2	1	0	2	4	2	0	1	1	1	14	(good)
15	Dallanora, A.	2012	10.1111/j.1741-2358.2011.00574.x	1	2	NA	0	2	3	2	0	1	0	0	11	(good)
16	De Stefano, A.	2022	10.1080/08869634.2020.1801013	1	2	NA	0	2	4	2	2	2	0	0	15	(outstanding)
17	Ebrahimi, M	2011	10.5681/joddd.2011.028	1	2	NA	0	2	4	2	0	0	0	0	11	(good)
18	Fernandes Azevedo, A.	2018	10.1080/08869634.2017.1361053	1	2	NA	0	2	3	2	2	2	0	0	14	(good)
19	Feteih, R.	2006	10.1186/1746-160X-2-25	0	2	NA	0	2	4	2	0	0	0	1	11	(good)
20	Figueiredo Ribeiro, D.	2020	PMID: 32788004	1	2	NA	0	2	3	2	0	0	0	0	10	(good)
21	Fonseca, F.	2022	10.1080/08869634.2022.2091099	1	2	1	0	2	4	2	0	0	0	1	13	(good)
22	Franco-Micheloni, A.	2015	10.11607/ofph.1262	1	2	NA	0	2	4	2	2	2	1	1	17	(outstanding)
23	Gesch, D.	2002	PMID: 15000638	1	2	1	0	2	4	2	0	0	0	0	12	(good)
24	Gonçalves, D.	2010	PMID: 20664828	1	2	1	0	2	4	2	0	0	1	1	14	(good)
25	Graue, A.	2016	10.1080/00016357.2016.1191086	1	2	NA	0	2	3	2	1	1	0	0	12	(good)
26	Habib, S.	2015	10.1016/j.sdentj.2014.11.009	1	2	1	0	2	4	2	0	0	0	0	12	(good)
27	Hadler-Olsen, E.	2021	10.1002/cre2.463	1	2	1	0	2	4	2	0	0	1	1	14	(good)
28	Hongxing, L.	2016	10.1111/joor.12366	1	2	2	0	2	4	2	0	0	1	1	15	(outstanding)
29	Isong, U.	2008	PMID: 19090404	1	2	2	0	2	4	2	0	0	1	1	15	(outstanding)
30	Jomhawi, J.	2021	10.5005/jp-journals-10005-1939	0	2	NA	0	2	3	2	1	1	0	0	11	(good)
31	Júnior, P.	2019	10.1371/journal.pone.0205874	1	2	NA	0	2	4	2	2	2	1	1	17	(outstanding)
32	Jussila, P.	2017	10.11607/ofph.1773	1	2	NA	0	2	4	2	2	2	0	0	15	(outstanding)
33	Karthik, R.	2017	10.4103/jispcd.JISPCD_146_17	0	2	1	0	2	4	2	0	1	0	0	12	(good)
34	Khan, K.	2020	10.1111/joor.12923	1	2	1	0	2	4	2	0	0	1	1	14	(good)
35	Kmeid, E.	2020	10.1186/s13005-020-00234-2	0	2	1	0	2	4	2	0	1	1	1	14	(good)
36	Lei, J.	2016	10.1179/2151090315Y.0000000021	1	2	1	0	2	4	2	0	1	0	0	13	(good)
37	Loster, J.	2017	10.1111/jopr.12414	1	2	NA	0	2	3	2	2	2	0	0	14	(good)
38	Marpaung, C.	2018	10.1155/2018/5053709	0	2	1	0	2	4	2	0	0	0	0	11	(good)
39	Marpaung, C.	2018	10.1111/cdoe.12382	1	2	1	0	2	4	2	0	0	1	1	14	(good)

40	Medeiros, R.	2020	10.1590/1678-7757-2020-0445	1	2	NA	0	2	3	2	2	2	0	1	15	(outstanding)
41	Mello, V.	2014	10.1590/0103-6440201302250	1	2	NA	0	2	3	2	1	2	1	1	15	(outstanding)
42	Mendiburu-Zavala, C.	2020	10.24875/BMHIM.20000002	0	2	NA	0	2	3	2	0	0	0	1	10	(good)
43	Moyaho-Bernal, A.	2010	PMID: 21638964	1	2	NA	0	2	3	2	2	2	0	1	15	(outstanding)
44	Natu, V.	2018	10.1111/joor.12692	1	2	1	0	2	3	2	0	1	0	1	13	(good)
45	Nekora-Azak, A.	2006	10.1111/j.1365-2842.2006.01543.x	0	2	1	0	2	3	2	0	0	0	1	11	(good)
46	Nilsson, I.-M	2005	PMID: 15895837	1	2	2	0	2	4	2	0	0	0	0	13	(good)
47	Nourallah, H.	1995	10.1111/j.1365-2842.1995.tb00783.x	0	2	NA	0	2	3	2	0	1	0	0	10	(good)
48	Oliveira, A.	2006	10.1590/S1806-83242006000100002	1	2	1	0	2	4	2	0	1	0	0	13	(good)
49	Özdinç, S.	2020	10.1080/08869634.2018.1513442	1	2	1	0	2	3	2	0	1	1	1	14	(good)
50	Paduano, S.	2020	10.1080/08869634.2018.1556893	0	2	NA	0	2	3	2	2	2	1	1	15	(outstanding)
51	Pedroni, C.	2003	10.1046/j.1365-2842.2003.01010.x	0	2	1	0	2	3	2	0	1	0	0	11	(good)
52	Perrotta, S.	2019	10.1111/joor.12794	1	2	NA	0	2	4	2	0	0	1	1	13	(good)
53	Pow, E.	2001	PMID: 11575193	0	2	1	0	2	4	2	0	0	0	0	11	(good)
54	Prakash, J.	2022	PMID: 35945831	0	2	NA	0	2	3	2	0	0	0	1	10	(good)
55	Progiante, P.	2015	10.11607/ijp.4026	1	2	NA	0	2	4	2	2	2	0	0	15	(outstanding)
56	Qvintus, V.	2020	10.1080/00016357.2020.1746395	1	2	2	0	2	4	2	0	0	1	1	15	(outstanding)
57	Ramírez-Caro, S.	2015	10.15446/rsap.v17n2.27958	0	2	NA	0	2	3	2	2	2	0	0	13	(good)
58	Rantala, M.	2023	PMID: 14737875	1	2	1	0	2	4	2	1	1	0	0	14	(good)
59	Sampaio, N.	2017	10.1590/2317-1782/20162016114	1	2	1	0	2	3	2	0	1	1	1	14	(good)
60	Song, H.-S.	2018	10.1371/journal.pone.0191336	1	2	2	0	2	4	2	0	0	1	1	15	(outstanding)
61	Srivastava, K.	2021	10.1186/s12903-021-01578-0	1	2	NA	0	2	4	2	0	1	1	1	14	(good)
62	Storm, C.	2006	10.1080/00016350600801915	0	2	1	0	2	4	2	0	1	1	1	14	(good)
63	Talaat, W.	2018	10.1016/j.oooo.2017.11.012	0	2	1	0	2	4	2	0	1	0	0	12	(good)
64	Taneja, P.	2019	10.5005/jp-journals-10005-1689	0	2	2	0	2	3	2	0	1	1	1	14	(good)
65	Tecco, S.	2011	10.1179/crn.2011.010	0	2	NA	0	2	4	2	2	2	1	1	16	(outstanding)
66	Tecco, S.	2019	10.1080/08869634.2017.1391963	1	2	NA	0	2	4	2	1	1	1	1	15	(outstanding)
67	Vainionpää, R.	2019	10.1080/00016357.2018.1535660	1	2	NA	0	2	3	2	2	2	0	0	14	(good)
68	Verdonck, A.	1994	10.1111/j.1365-2842.1994.tb01184.x	0	2	NA	0	2	4	2	0	1	0	0	11	(good)
69	Wieckiewicz, M.	2020	10.11607/ofph.2386	1	2	NA	0	2	4	2	2	2	1	1	17	(outstanding)

70	Wu, J.	2021	10.1186/s12903-021-01916-2	0	2	1	0	2	4	2	0	1	0	0	12	(good)
71	Wu, N.	2010	10.1007/s00056-010-1004-x	0	2	NA	0	2	4	2	2	2	1	1	16	(outstanding)
72	Yasuda, E.	2015	10.13075/ijomeh.1896.00524	1	2	NA	0	2	3	2	0	0	0	0	10	(good)
73	Yekkalam, N.	2014	10.3109/00016357.2013.860620	1	2	2	0	2	4	2	0	1	0	0	14	(good)
74	Yu, Q.	2015	10.1007/s00420-015-1018-1	1	2	NA	0	2	4	2	2	2	1	1	17	(outstanding)
75	Adegbiji, W.	2021	10.1177/0300060521996517	0	2	NA	0	2	3	2	0	1	0	0	10	(good)
76	Friedman Rubin, P.	2018	10.1080/08869634.2017.1331784	1	2	NA	0	2	3	2	2	2	0	1	15	(outstanding)
77	Lung, J.	2018	10.1111/jicd.12341.	1	2	NA	0	2	4	2	2	2	0	0	15	(outstanding)
78	Zwiri, A.	2016	10.1179/2151090315Y.0000000007	1	2	NA	0	2	4	2	0	1	0	0	12	(good)

NA: Not applicable
