# Supplementary material for: A Meta-Analysis of the Global Prevalence of Temporomandibular Disorders
Source: J Clin Med. 2024 Feb 28;13(5):1365. doi: 10.3390/jcm13051365 (PMC10931584; doi:10.3390/jcm13051365)
Supplement: Supplementary file 1 [file jcm-13-01365-s001.zip › Supplementary Material S2.pdf]

1. Akhter, R., Hassan, N. M. M., Ohkubo, R., Tsukazaki, T., Aida, J., & Morita, M. (2008). The relationship between jaw injury, third molar removal, and orthodontic treatment and TMD symptoms in university students in Japan. Journal of Orofacial Pain, 22(1), 50–56.

2. Alketbi, N., & Talaat, W. (2022). Prevalence and characteristics of referred pain in patients diagnosed with temporomandibular disorders according to the Diagnostic Criteria for Temporomandibular Disorders (DC/TMD) in Sharjah, United Arab Emirates. F1000Research. https://doi.org/10.12688/f1000research.109696.2

3. Al-Khotani, A., Naimi-Akbar, A., Albadawi, E., Ernberg, M., Hedenberg-Magnusson, B., & Christidis, N. (2016). Prevalence of diagnosed temporomandibular disorders among Saudi Arabian children and adolescents. The Journal of Headache and Pain, 17(1), 41. https://doi.org/10.1186/s10194-016-0642-9

4. Alkhubaizi, Q., Khalaf, M. E., & Faridoun, A. (2022). Prevalence of Temporomandibular Disorder-Related Pain among Adults Seeking Dental Care: A Cross-Sectional Study. International Journal of Dentistry, 2022, e3186069. https://doi.org/10.1155/2022/3186069

5. Alrashdan, M. S., Nuseir, A., & AL-Omiri, M. K. (2019). Prevalence and correlations of temporomandibular disorders in Northern Jordan using diagnostic criteria axis I. Journal of Investigative and Clinical Dentistry, 10(2), e12390. https://doi.org/10.1111/jicd.12390

6. Bahrani, F., Ghadiri, P., & Vojdani, M. (2012). Comparison of temporomandibular disorders in Iranian dental and nondental students. The Journal of Contemporary Dental Practice, 13(2), 173–177. https://doi.org/10.5005/jp-journals-10024-1116

7. Banafa, A., Suominen, A. L., & Sipilä, K. (2020). Factors associated with signs of temporomandibular pain: an 11-year-follow-up study on Finnish adults. Acta Odontologica Scandinavica, 78(1), 57–63. https://doi.org/10.1080/00016357.2019.1650955

8. Barbosa, C., Manso, M. C., Reis, T., Soares, T., Gavinha, S., & Ohrbach, R. (2021). Are oral overuse behaviours associated with painful temporomandibular disorders? A cross-sectional study in Portuguese university students. Journal of Oral Rehabilitation, 48(10), 1099–1108. https://doi.org/10.1111/joor.13226

9. Bertoli, F. M. de P., Bruzamolin, C. D., Pizzatto, E., Losso, E. M., Brancher, J. A., & Souza, J. F. de. (2018). Prevalence of diagnosed temporomandibular disorders: A cross-sectional study in Brazilian adolescents. PLOS ONE, 13(2), e0192254. https://doi.org/10.1371/journal.pone.0192254

10. Camacho, J. G. D. D., Oltramari-Navarro, P. V. P., Navarro, R. de L., Conti, A. C. de C. F., Conti, M. R. de A., Marchiori, L. L. de M., & Fernandes, K. B. P. (2014). Signs and symptoms of Temporomandibular Disorders in the elderly. CoDAS, 26, 76–80. https://doi.org/10.1590/S2317-17822014000100011

11. Campos, J. A. D. B., Carrascosa, A. C., Bonafé, F. S. S., & Maroco, J. (2014). Epidemiology of severity of temporomandibular disorders in Brazilian women. Journal of Oral & Facial Pain and Headache, 28(2), 147–152. https://doi.org/10.11607/ofph.1194

12. Carlsson, G. E., Ekbäck, G., Johansson, A., Ordell, S., & Unell, L. (2014). Is there a trend of decreasing prevalence of TMD-related symptoms with ageing among the elderly? Acta Odontologica Scandinavica, 72(8), 714–720. https://doi.org/10.3109/00016357.2014.898787

13. Choi, Y.-S., Choung, P.-H., Moon, H.-S., & Kim, S.-G. (2002). Temporomandibular disorders in 19-year-old Korean men. Journal of Oral and Maxillofacial Surgery, 60(7), 797–803. https://doi.org/10.1053/joms.2002.33249

14. Ciancaglini, R., & Radaelli, G. (2001). The relationship between headache and symptoms of temporomandibular disorder in the general population. Journal of Dentistry, 29(2), 93–98. https://doi.org/10.1016/S0300-5712(00)00042-7

15. Dallanora, A. F., Grasel, C. E., Heine, C. P., Demarco, F. F., Pereira-Cenci, T., Presta, A. A., & Boscato, N. (2012). Prevalence of temporomandibular disorders in a population of complete denture wearers. Gerodontology, 29(2), e865–e869. https://doi.org/10.1111/j.1741-2358.2011.00574.x

16. De Stefano, A. A., Guercio-Mónaco, E., Uzcátegui, A., Boboc, A. M., Barbato, E., & Galluccio, G. (2022). Temporomandibular disorders in Venezuelan and Italian adolescents. CRANIO®, 40(6), 517–523. https://doi.org/10.1080/08869634.2020.1801013

17. Ebrahimi, M., Dashti, H., Mehrabkhani, M., Arghavani, M., & Daneshvar-Mozafari, A. (2011). Temporomandibular Disorders and Related Factors in a Group of Iranian Adolescents: A Cross-sectional Survey. Journal of Dental Research, Dental Clinics, Dental Prospects, 5(4), 123–127. https://doi.org/10.5681/joddd.2011.028

18. Fernandes Azevedo, A. B., Câmara-Souza, M. B., Dantas, I. de S., de Resende, C. M. B. M., & Barbosa, G. A. S. (2018). Relationship between anxiety and temporomandibular disorders in dental students. CRANIO®, 36(5), 300–303. https://doi.org/10.1080/08869634.2017.1361053

19. Feteih, R. M. (2006). Signs and symptoms of temporomandibular disorders and oral parafunctions in urban Saudi arabian adolescents: a research report. Head & Face Medicine, 2(1), 25. https://doi.org/10.1186/1746-160X-2-25

20. Figueiredo Ribeiro, D. C., Ferreira Gradella, C. M., Franco Rocha Rodrigues, L. L., Abanto, J., & Oliveira, L. B. (2020). The Impact of Temporomandibular Disorders on the Oral Health-Related Quality of Life of Brazilian Children: A Cross-Sectional Study. Journal of Dentistry for Children, 87(2), 103–109.

21. Fonseca, F. F., Politti, F., Cunha, T., Leonardis, M., Carvalho, L., de Paula Gomes, C. A. F., & Biasotto-Gonzalez, D. A. (2022). Prevalence of signs and symptoms of temporomandibular disorder in the metropolitan region of Rio De Janeiro: A population-based cross-sectional study. CRANIO®, 0(0), 1–7. https://doi.org/10.1080/08869634.2022.2091099

22. Franco-Micheloni, A. L., Fernandes, G., de Godoi Gonçalves, D. A., & Camparis, C. M. (2015). Temporomandibular Disorders in a Young Adolescent Brazilian Population: Epidemiologic Characterization and Associated Factors. Journal of Oral & Facial Pain and Headache, 29(3), 242–249. https://doi.org/10.11607/ofph.1262

23. Gesch, D., Bernhardt, O., Alte, D., Schwahn, C., Kocher, T., John, U., & Hensel, E. (2004). Prevalence of signs and symptoms of temporomandibular disorders in an urban and rural German population: results of a population-based Study of Health in Pomerania. Quintessence International (Berlin, Germany: 1985), 35(2), 143–150.

24. Gonçalves, D. A. de G., Dal Fabbro, A. L., Campos, J. A. D. B., Bigal, M. E., & Speciali, J. G. (2010). Symptoms of temporomandibular disorders in the population: an epidemiological study. Journal of Orofacial Pain, 24(3), 270–278.

25. Graue, A. M., Jokstad, A., Assmus, J., & Skeie, M. S. (2016). Prevalence among adolescents in Bergen, Western Norway, of temporomandibular disorders according to the DC/TMD criteria and examination protocol. Acta Odontologica Scandinavica, 74(6), 449–455. https://doi.org/10.1080/00016357.2016.1191086

26. Habib, S. R., Al Rifaiy, M. Q., Awan, K. H., Alsaif, A., Alshalan, A., & Altokais, Y. (2015). Prevalence and severity of temporomandibular disorders among university students in Riyadh. The Saudi Dental Journal, 27(3), 125–130. https://doi.org/10.1016/j.sdentj.2014.11.009

27. Hadler-Olsen, E., Thon, E., Holde, G. E., Jönsson, B., Oscarson, N., & Tillberg, A. (2021). Temporomandibular disorders in an adult population in northern Norway: A cross-sectional study. Clinical and Experimental Dental Research, 7(6), 1144–1153. https://doi.org/10.1002/cre2.463

28. Hongxing, L., Astrøm, A. N., List, T., Nilsson, I.-M., & Johansson, A. (2016). Prevalence of temporomandibular disorder pain in Chinese adolescents compared to an age-matched Swedish population. Journal of Oral Rehabilitation, 43(4), 241–248. https://doi.org/10.1111/joor.12366

29. Isong, U., Gansky, S. A., & Plesh, O. (2008). Temporomandibular Joint and Muscle Disorder-type Pain in US Adults: The National Health Interview Survey. Journal of orofacial pain, 22(4), 317–322.

30. Jomhawi, J. M., Elsamarneh, A. M., & Hassan, A. M. (2021). Prevalence of Temporomandibular Disorder among Schoolchildren in Jordan. International Journal of Clinical Pediatric Dentistry, 14(2), 304–310. https://doi.org/10.5005/jp-journals-10005-1939

31. Júnior, P. C. de M., Aroucha, J. M. C. N. L., Arnaud, M., Lima, M. G. de S., Gomes, S. G. F., Ximenes, R., et al. (2019). Prevalence of TMD and level of chronic pain in a group of Brazilian adolescents. PLOS ONE, 14(2), e0205874. https://doi.org/10.1371/journal.pone.0205874

32. Jussila, P., Kiviahde, H., Näpänkangas, R., Päkkilä, J., Pesonen, P., Sipilä, K., et al. (2017). Prevalence of Temporomandibular Disorders in the Northern Finland Birth Cohort 1966. Journal of Oral & Facial Pain and Headache, 31(2), 159–164. https://doi.org/10.11607/ofph.1773

33. Karthik, R., Hafila, M. I. F., Saravanan, C., Vivek, N., Priyadarsini, P., & Ashwath, B. (2017). Assessing Prevalence of Temporomandibular Disorders among University Students: A Questionnaire Study. Journal of International Society of Preventive & Community Dentistry, 7(Suppl 1), S24–S29. https://doi.org/10.4103/jispcd.JISPCD_146_17

34. Khan, K., Muller-Bolla, M., Anacleto Teixeira Junior, O., Gornitsky, M., Guimarães, A. S., & Velly, A. M. (2020). Comorbid conditions associated with painful temporomandibular disorders in adolescents from Brazil, Canada and France: A cross-sectional study. Journal of Oral Rehabilitation, 47(4), 417–424. https://doi.org/10.1111/joor.12923

35. Kmeid, E., Nacouzi, M., Hallit, S., & Rohayem, Z. (2020). Prevalence of temporomandibular joint disorder in the Lebanese population, and its association with depression, anxiety, and stress. Head & Face Medicine, 16(1), 19. https://doi.org/10.1186/s13005-020-00234-2

36. Lei, J., Fu, J., Yap, A. U. J., & Fu, K.-Y. (2016). Temporomandibular disorders symptoms in Asian adolescents and their association with sleep quality and psychological distress. CRANIO®, 34(4), 242–249. https://doi.org/10.1179/2151090315Y.0000000021

37. Loster, J. E., Osiewicz, M. A., Groch, M., Ryniewicz, W., & Wieczorek, A. (2017). The Prevalence of TMD in Polish Young Adults. Journal of Prosthodontics, 26(4), 284–288. https://doi.org/10.1111/jopr.12414

38. Marpaung, C., Lobbezoo, F., & van Selms, M. K. A. (2018). Temporomandibular Disorders among Dutch Adolescents: Prevalence and Biological, Psychological, and Social Risk Indicators. Pain Research and Management, 2018, e5053709. https://doi.org/10.1155/2018/5053709

39. Marpaung, C., van Selms, M. K. A., & Lobbezoo, F. (2018). Prevalence and risk indicators of pain-related temporomandibular disorders among Indonesian children and adolescents. Community Dentistry and Oral Epidemiology, 46(4), 400–406. https://doi.org/10.1111/cdoe.12382

40. Medeiros, R. A. D., Vieira, D. L., Silva, E. V. F. D., Rezende, L. V. M. D. L., Santos, R. W. D., & Tabata, L. F. (2020). Prevalence of symptoms of temporomandibular disorders, oral behaviors, anxiety, and depression in Dentistry students during the period of social isolation due to COVID-19. Journal of Applied Oral Science, 28, e20200445. https://doi.org/10.1590/1678-7757-2020-0445

41. Mello, V. V. C. de, Barbosa, A. C. da S., Morais, M. P. L. de A., Gomes, S. G. F., Vasconcelos, M. M. V. B., & Caldas Júnior, A. de F. (2014). Temporomandibular Disorders in a Sample Population of the Brazilian Northeast. Brazilian Dental Journal, 25, 442–446. https://doi.org/10.1590/0103-6440201302250

42. Mendiburu-Zavala, C. E., Castillero-Rosas, A. S., Lugo-Ancona, P. E., & Carrillo-Mendiburu, J. (2020). Disfunción temporomandibular y depresión en adolescentes de ascendencia maya. Boletín Médico del Hospital Infantil de México, 77(3). https://doi.org/10.24875/BMHIM.20000002

43. Moyaho-Bernal, A., Lara-Muñoz, M. D. C., Espinosa-De Santillana, I., & Etchegoyen, G. (2010). Prevalence of signs and symptoms of temporomandibular disorders in children in the State of Puebla, Mexico, evaluated with the research diagnostic criteria for temporomandibular disorders (RDC/TMD). Acta odontologica latinoamericana: AOL, 23(3), 228–233.

44. Natu, V. P., Yap, A. U.-J., Su, M. H., Irfan Ali, N. M., & Ansari, A. (2018). Temporomandibular disorder symptoms and their association with quality of life, emotional states and sleep quality in South-East Asian youths. Journal of Oral Rehabilitation, 45(10), 756–763. https://doi.org/10.1111/joor.12692

45. Nekora-Azak, A., Evlioglu, G., Ordulu, M., & İşsever, H. (2006). Prevalence of symptoms associated with temporomandibular disorders in a Turkish population. Journal of Oral Rehabilitation, 33(2), 81–84. https://doi.org/10.1111/j.1365-2842.2006.01543.x

46. Nilsson, I.-M., List, T., & Drangsholt, M. (2005). Prevalence of temporomandibular pain and subsequent dental treatment in Swedish adolescents. Journal of Orofacial Pain, 19(2), 144–150.

47. Nourallah, H., & Johansson, A. (1995). Prevalence of signs and symptoms of temporomandibular disorders in a young male Saudi population. Journal of Oral Rehabilitation, 22(5), 343–347. https://doi.org/10.1111/j.1365-2842.1995.tb00783.x

48. Oliveira, A. S. de, Dias, E. M., Contato, R. G., & Berzin, F. (2006). Prevalence study of signs and symptoms of temporomandibular disorder in Brazilian college students. Brazilian Oral Research, 20, 3–7. https://doi.org/10.1590/S1806-83242006000100002

49. Özdinç, S., Ata, H., Selçuk, H., Can, H. B., Sermenli, N., & Turan, F. N. (2020). Temporomandibular joint disorder determined by Fonseca anamnestic index and associated factors in 18- to 27-year-old university students. CRANIO®, 38(5), 327–332. https://doi.org/10.1080/08869634.2018.1513442

50. Paduano, S., Bucci, R., Rongo, R., Silva, R., & Michelotti, A. (2020). Prevalence of temporomandibular disorders and oral parafunctions in adolescents from public schools in Southern Italy. CRANIO®, 38(6), 370–375. https://doi.org/10.1080/08869634.2018.1556893

51. Pedroni, C. R., De Oliveira, A. S., & Guaratini, M. I. (2003). Prevalence study of signs and symptoms of temporomandibular disorders in university students. Journal of Oral Rehabilitation, 30(3), 283–289. https://doi.org/10.1046/j.1365-2842.2003.01010.x

52. Perrotta, S., Bucci, R., Simeon, V., Martina, S., Michelotti, A., & Valletta, R. (2019). Prevalence of malocclusion, oral parafunctions and temporomandibular disorder-pain in Italian schoolchildren: An epidemiological study. Journal of Oral Rehabilitation, 46(7), 611–616. https://doi.org/10.1111/joor.12794

53. Pow, E. H., Leung, K. C., & McMillan, A. S. (2001). Prevalence of symptoms associated with temporomandibular disorders in Hong Kong Chinese. Journal of Orofacial Pain, 15(3), 228–234.

54. Prakash, J., Ranvijay, K., Devi, L. S., Shenoy, M., Abdul, N. S., Shivakumar, G. C., & Gupta, P. (2022). Assessment of Symptoms Associated with Temporomandibular Dysfunction and Bruxism among Elderly Population: An Epidemiological Survey. The Journal of Contemporary Dental Practice, 23(4), 393–398.

55. Progiante, P. S., Pattussi, M. P., Lawrence, H. P., Goya, S., Grossi, P. K., & Grossi, M. L. (2015). Prevalence of Temporomandibular Disorders in an Adult Brazilian Community Population Using the

Research Diagnostic Criteria (Axes I and II) for Temporomandibular Disorders (The Maringá Study). The International Journal of Prosthodontics, 28(6), 600–609. https://doi.org/10.11607/ijp.4026

56. Qvintus, V., Sipilä, K., Le Bell, Y., & Suominen, A. L. (2020). Prevalence of clinical signs and pain symptoms of temporomandibular disorders and associated factors in adult Finns. Acta Odontologica Scandinavica, 78(7), 515–521. https://doi.org/10.1080/00016357.2020.1746395

57. Ramírez-Caro, S. N., Espinosa de Santillana, I. A., & Muñoz-Quintana, G. (2015). [Prevalence of temporomandibular disorders in Mexican children with mixed dentition]. Revista De Salud Publica (Bogota, Colombia), 17(2), 289–299. https://doi.org/10.15446/rsap.v17n2.27958

58. Rantala, M. A., Ahlberg, J., Suvinen, T. I., Savolainen, A., & Könönen, M. (2003). Symptoms, signs, and clinical diagnoses according to the research diagnostic criteria for temporomandibular disorders among Finnish multiprofessional media personnel. Journal of Orofacial Pain, 17(4), 311–316.

59. Sampaio, N. de M., Oliveira, M. C., Ortega, A. de O., Santos, L. de B., & Alves, T. D. B. (2017). Temporomandibular disorders in elderly individuals: the influence of institutionalization and sociodemographic factors. CoDAS, 29, e20160114. https://doi.org/10.1590/2317-1782/20162016114

60. Song, H.-S., Shin, J.-S., Lee, J., Lee, Y. J., Kim, M., Cho, J.-H., et al. (2018). Association between temporomandibular disorders, chronic diseases, and ophthalmologic and otolaryngologic disorders in Korean adults: A cross-sectional study. PLOS ONE, 13(1), e0191336. https://doi.org/10.1371/journal.pone.0191336

61. Srivastava, K. C., Shrivastava, D., Khan, Z. A., Nagarajappa, A. K., Mousa, M. A., Hamza, M. O., et al. (2021). Evaluation of temporomandibular disorders among dental students of Saudi Arabia using Diagnostic Criteria for Temporomandibular Disorders (DC/TMD): a cross-sectional study. BMC Oral Health, 21(1), 211. https://doi.org/10.1186/s12903-021-01578-0

62. Storm, C., & Wänman, A. (2006). Temporomandibular disorders, headaches, and cervical pain among females in a Sami population. Acta Odontologica Scandinavica, 64(5), 319–325. https://doi.org/10.1080/00016350600801915

63. Talaat, W. M., Adel, O. I., & Al Bayatti, S. (2018). Prevalence of temporomandibular disorders discovered incidentally during routine dental examination using the Research Diagnostic Criteria for Temporomandibular Disorders. Oral Surgery, Oral Medicine, Oral Pathology and Oral Radiology, 125(3), 250–259. https://doi.org/10.1016/j.oooo.2017.11.012

64. Taneja, P., Nagpal, R., Marya, C. M., Kataria, S., Sahay, V., & Goyal, D. (2019). Temporomandibular Disorders among Adolescents of Haryana, India: A Cross-sectional Study. International Journal of Clinical Pediatric Dentistry, 12(6), 500–506. https://doi.org/10.5005/jp-journals-10005-1689

65. Tecco, S., Crincoli, V., Di Bisceglie, B., Saccucci, M., Macrí, M., Polimeni, A., & Festa, F. (2011). Signs and Symptoms of Temporomandibular Joint Disorders in Caucasian Children and Adolescents. CRANIO®, 29(1), 71–79. https://doi.org/10.1179/crn.2011.010

66. Tecco, S., Nota, A., Caruso, S., Primozic, J., Marzo, G., Baldini, A., & Gherlone, E. F. (2019). Temporomandibular clinical exploration in Italian adolescents. CRANIO®, 37(2), 77–84. https://doi.org/10.1080/08869634.2017.1391963

67. Vainionpää, R., Kinnunen, T., Pesonen, P., Laitala, M.-L., Anttonen, V., & Sipilä, K. (2019). Prevalence of temporomandibular disorders (TMD) among Finnish prisoners: cross-sectional clinical study. Acta Odontologica Scandinavica, 77(4), 264–268. https://doi.org/10.1080/00016357.2018.1535660

68. Verdonck, A., Takada, K., Kitai, N., Kuriama, R., Yasuda, Y., Carels, C., & Sakuda, M. (1994). The prevalence of cardinal TMJ dysfunction symptoms and its relationship to occlusal factors in Japanese female adolescents. Journal of Oral Rehabilitation, 21(6), 687–697. https://doi.org/10.1111/j.1365-2842.1994.tb01184.x

69. Wieckiewicz, M., Grychowska, N., Nahajowski, M., Hnitecka, S., Kempiak, K., Charemska, K., et al. (2020). Prevalence and Overlaps of Headaches and Pain-Related Temporomandibular Disorders Among the Polish Urban Population. Journal of Oral & Facial Pain and Headache, 34(1), 31–39. https://doi.org/10.11607/ofph.2386

70. Wu, J., Huang, Z., Chen, Y., Chen, Y., Pan, Z., & Gu, Y. (2021). Temporomandibular disorders among medical students in China: prevalence, biological and psychological risk factors. BMC Oral Health, 21(1), 549. https://doi.org/10.1186/s12903-021-01916-2

71. Wu, N., & Hirsch, C. (2010). Temporomandibular Disorders in German and Chinese Adolescents. Journal of Orofacial Orthopedics / Fortschritte der Kieferorthopädie, 71(3), 187–198. https://doi.org/10.1007/s00056-010-1004-x

72. Yasuda, E., Honda, K., Hasegawa, Y., Matsumura, E., Fujiwara, M., Hasegawa, M., & Kishimoto, H. (2015). Prevalence of temporomandibular disorders among junior high school students who play wind instruments. International Journal of Occupational Medicine and Environmental Health, 29(1), 69–76. https://doi.org/10.13075/ijomeh.1896.00524

73. Yekkalam, N., & Wänman, A. (2014). Prevalence of signs and symptoms indicative of temporomandibular disorders and headaches in 35-, 50-, 65- and 75-year-olds living in Västerbotten, Sweden. Acta Odontologica Scandinavica, 72(6), 458–465. https://doi.org/10.3109/00016357.2013.860620

74. Yu, Q., Liu, Y., Chen, X., Chen, D., Xie, L., Hong, X., et al. (2015). Prevalence and associated factors for temporomandibular disorders in Chinese civilian pilots. International Archives of Occupational and Environmental Health, 88(7), 905–911. https://doi.org/10.1007/s00420-015-1018-1
