# Supplementary material for: A Meta-Analysis of the Global Prevalence of Temporomandibular Disorders
Source: J Clin Med. 2024 Feb 28;13(5):1365. doi: 10.3390/jcm13051365 (PMC10931584; doi:10.3390/jcm13051365)
Supplement: Supplementary file 1 [file jcm-13-01365-s001.zip › Supplementary Material S3.pdf]

List of abbreviations of the names of statistical measures:
N – sample size;
n – group size;
α – significance level;
k – number of studies;
p – the p-value of the statistical test;
Mdn – median;
Min – minimum value;
Max – maximum value;
Q1 – the first quartile (25%);
Q3 – the third quartile (75%);
Q – the *Q* statistic;
df – degrees of freedom;
I^2 – total variability;
CI 95% – confidence interval 95%;
τ_{rc} – Kendall's tau rank correlation;
t – *t*-Student test statistic;
df – degrees of freedom;
AIC – Akaike's Information Criterion;
logLik – log likelihood;
Δ – evaluation metric delta compared to the best model;
τ^2 – amount of total (residual) heterogeneity;
I^2 – (residual) heterogeneity / total (unaccounted) variability;
H^2 – total (unaccounted) variability / sampling variability;
R^2 – explained amount of the heterogeneity;
Q – Cochran's Q statistic;
OR – the odds ratios;

The present meta-analysis aims to evaluate the proportion of individuals with TMDs across various studies, considering factors such as geographical region, patient age and sample size. To the best of our knowledge, there was no meta-analysis on the prevalence of TMDs according to geographical region.

1. Methods and Materials

We conducted a systematic search and review of TMDs prevalence based on surveys conducted from 1 January 1994 to 1 December 2022. The starting date was chosen because it was 2 years after the introduction of the RDC/TMD questionnaire [1], (which could have allowed for standardised research on TMDs). Additionally it is the date of the introduction of the second standardised FAI survey protocol [2].

We searched PubMed (National Library of Medicine) [3–6] from 9 January to 11 June 2023 for publications using the following MeSH (Medical Subject Heading) terms: temporomandibular disorders AND prevalence, temporomandibular disorders AND epidemiology, temporomandibular disorders AND population. Based on the work of Valesan et al. no restrictions on age, gender or language of publication were applied. Both painful and non-painful TMDs were accepted [7].

The search yielded 6984 articles on the incidence of TMDs. The titles, abstract of each publication were reviewed and articles that were population-based studies were included. Studies were excluded if they were case studies or involved animal studies. In this step, 78 studies that fell into the good ([8–62]) and outstanding ([63–85]) groups on the basis of TOS were assessed (Supplementary Material S1).

During the pre-final step of the evaluation of the 78 studies, it was noted that only 2 studies were from the African population [61,84] and 1 study was from the Australian population [85]. Due to the main purpose of the study - meta-analysis of the prevalence of TMDs in relation to continents. Due to the inability to perform a meta-analysis of individual studies, it was decided to omit these studies. One influential case [86] was identified during data pre-processing, and study [62] was removed from further analysis. Finally, 74 studies that fell into the good ([8–60]) and excellent ([63–83]) groups on the basis of the TOS (Figure S1, Supplementary Material S2) were included, which analysed 80 populations (studies that analysed more than one population): Khan et al. analysis of TMDs incidence in Europe, North America and South America [8], Wu et al. European and Asian populations [49] and Hongxing et al. European and Asian populations [75], De Stefano et al. European and South American populations [68]). In order to determine the age of onset of TMDs in each continent, an age classification was made as follows: "up to 18 yrs (years)", "18-60 yrs" and "60+ yrs" [87]. In addition, a study by Yekkalam and Wänman on the Swedish population was divided into two age groups ("18-60 yrs" and "60+ yrs") for the purpose of meta-analysis [60].

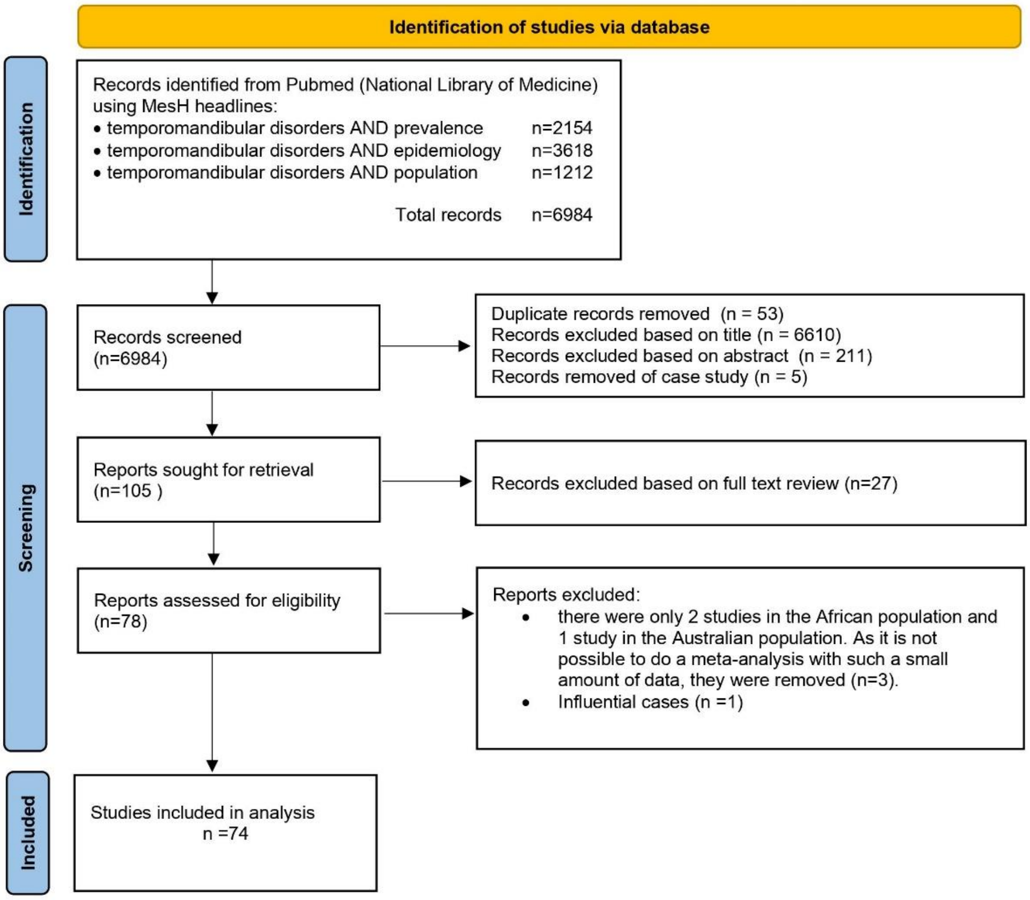

Figure S1. PRISMA flow diagram.

1.1.Characteristic of the sample

The meta-analysis aimed to pool results from k=80 studies, comprising a total of 172,239 observations, with 35,259 recorded events, focusing on the occurrence of Temporomandibular Disorders (TMDs) as a dichotomous dependent variable. The TMDs metric represented the number of individuals with TMDs occurrence relative to the total number of individuals in each study (a proportion meta-analysis without a control group).
The studies are classified based on several factors:

- geographical region: studies were categorized by the continent where the research was conducted, including Asia, Europe, South America, and North America.
- age groups: patient age is categorized into three distinct groups: younger patients (up to 18 yrs), young and middle-aged patients (18-60 yrs), older patients (over 60 yrs)
- sample size: includes the overall size of the sample and the gender distribution within the sample (number of males and females) (Table S1).

Table S1. Distribution of variables of the study sample

Characteristic	*N*	*Distribution*[1]
Continent:	80	
Asia		31 (39.8%)
Europe		26 (32.0%)
North America		5 (6.2%)
South America		18 (22.0%)
Age:	71	
18-60 yrs.		32 (45.0%)
60+ yrs.		5 (7.0%)
up to 18 yrs.		34 (48.0%)
Sample size	80	542 (245.0, 1,236.0)[2],<br>*Min* = 50.0<br>*Max* = 30 978.0
TMDs	80	156 (87.0, 390.0)<br>*Min* = 20.0<br>*Max* = 9624.0
Female group size	61	267 (135.0, 638.0) [2]<br>*Min* = 11.0<br>*Max* =17498.0
Male group size	61	206 (89.0, 534.0) [2]<br>*Min* = 17.0

Characteristic	*N*	*Distribution*[1]
		Max =15056.0

[1] *n* (%); [2]*Mdn*(*Q1*, *Q3*)

2. Statistical analysis

2.1.Significance level

The significance level of the statistical tests in this analysis was set at $\alpha = 0.05$.

2.2.Effect size estimation

Each study's proportion of TMDs was computed as the ratio of MDR occurrences to the sample size. Given the non-normal distribution of these proportions, they were transformed to log ratios or log odds. The corresponding sample variance was then estimated for each study. A random-effects model was applied to calculate the logit proportion across all studies, with heterogeneity (τ^2) evaluated using the DerSimonian-Laird estimator [88,89], its confidence interval was determined using the Jackson method [90].

2.3.Identifying outliers and influential cases

Outliers were detected by 'studentizing' the residuals from the random-effects model. In this scenario, any absolute residual with a z-score exceeding 2.0 was classified as an outlier.

To assess the influence of these outliers, we employed visual diagnostic techniques, specifically the leave-one-out method [86]. This process entailed examining each case in isolation, using metrics such as the externally standardized residual, DFFITS value, Cook's distance, and covariance ratio. Additionally, we evaluated the leave-one-out amount of (residual) heterogeneity, the leave-one-out test statistic for (residual) heterogeneity, and DFBETAS values.

2.4. Subgroup analysis

For a more granular understanding of heterogeneity, we conducted a subgroup analysis. Studies were stratified based on one or more factors under investigation. The procedure involved computing aggregate proportions and their corresponding 95% confidence intervals for each subgroup using a random-effects model. Both within-study and between-study variances were estimated in this process. The inverse variance method [91] was applied to establish the weight of individual studies.

Heterogeneity within subgroups was assessed using the I^2 statistic, the Q statistic, τ^2, and τ. The I^2 statistic and Q statistic were used to evaluate the percentage of total variation across studies due to heterogeneity rather than chance. The τ^2 and τ values, representing the estimated between-study variance and standard deviation respectively, provided further measures of heterogeneity within each subgroup. The Q statistic was also utilized to test for significant differences in effect size across the defined subgroups.

The results of the subgroup analysis were also visualized in the form of a forest plot.

2.5. Publication bayes

Publication bias was evaluated using a comprehensive, multi-step approach to provide a thorough and robust assessment. First, we constructed a funnel plot based on a random-effects model. This graphical representation provided an intuitive way to visually assess the presence of publication bias.

Next, we used the 'trim and fill' method, an iterative procedure developed by Duval and Tweedie [92,93], to adjust for potential publication bias in our meta-analysis. This method seeks to identify and 'fill' any gaps in the funnel plot, which represent potentially missing studies due to publication bias. The imputed studies were used to recalculate a corrected pooled effect size, providing an estimate of the effect size that might have been observed in the absence of publication bias.

Following the graphical assessment, we statistically evaluated funnel plot asymmetry using Egger's regression test [94]. This test uses a linear regression model with the study effect sizes as the response variable and their standard errors as the predictor.

Finally, we performed the rank correlation test established by Begg and Mazumdar [95] to investigate whether there was a correlation between the effect sizes (or outcomes) and their corresponding sample variances.

2.6. Statistical environment

Analyses were conducted using the R Statistical language (version 4.1.1; R Core Team, 2021) on Windows 10 Pro 64 bit (build 19045), using the packages *meta* (version 6.0.0; [96]), *weightr* (version 2.0.2; [97]), *report* (version 0.5.7; [98]), *metasens* (version 1.5.0; [99]), *metafor* (version 3.8.1; [100]), *ggplot2* (version 3.4.0; [101]), readxl (version 1.3.1; [102]), *dplyr* (version 1.1.2; [103]), *dmetar* (version 0.0.9000; [104]), and *scales* (version 1.2.1; [105]).

3. Results

3.1. Random effect model results for the sample

The summary proportion (the prevalence of TMDs) for the whole sample[1] was 0.34 with *CI 95%* [0.29, 0.39]. The $\tau^2 = 1.06$, *SE* = 0.41. The $\tau = 1.03$, *CI 95%* [0.83; 1.23] suggested a high level of variability between the studies' results that was not explained by sampling error alone. The $I^2 = 99.64\%$ statistic, *CI 95%* [99.6%; 99.7%] indicated that almost all of the observed variability in the effect sizes across studies can be attributed to true differences in effect size (heterogeneity) rather than just chance (sampling error). The $H^2 = 280.46$, *CI 95%* [16.29; 17.22] which was a large value suggesting a high level of unaccounted variability relative to the sampling variability. The results of heterogeneity test statistic Q (79)=22156.06, $p < 0.010$, further confirmed the presence of substantial heterogeneity across the studies.

While the meta-analysis provides a robust estimate of the prevalence of TMDs, the high heterogeneity and variability among the studies suggested that additional factors not included in the model may be influencing the results. These factors could include study design, participant characteristics, or other contextual variables.

3.2. Identifying outliers and influential cases

Of the 80 studies, *k*=6 were identified as outliers (with $z > 2.0$). Visualization of the leave-one-out approach in Figure S2 based on eight different metrics[2] showed that none of the outlier variables had an influential case.

[1] After transformation from log odds.

[2] The *dffits* value essentially indicates by how many standard deviations the predicted (average) effect or outcome for the case changes after the case is excluded from the model fit. The Cook distance *cook.d* can be interpreted as the Mahalanobis distance between the entire set of predicted values, once with the case included and once with the case excluded from model fitting. The covariance ratio *cov.r* was defined as the determinant of the variance-covariance matrix of the parameter estimates based on the data set without the case divided by the determinant of the variance-covariance matrix of the parameter estimates based on the full data set. Therefore, a value less than 1 indicates that removing the case results in more accurate estimates of the model coefficients. The amount of (residual) heterogeneity *tau2.del* is the estimated value of based on the data set without the case removed. This value is always equal to 0 for models with equal effects. Similarly, the leave-one-out test statistic of the test for (residual) heterogeneity *QE.del* was the value of the test statistic of the test for (residual) heterogeneity calculated based on the data set without the case. The diagonal elements of the *hat* matrix and the *weights* (in %) assigned to the observed effect sizes or outcomes during model fitting were also reported.

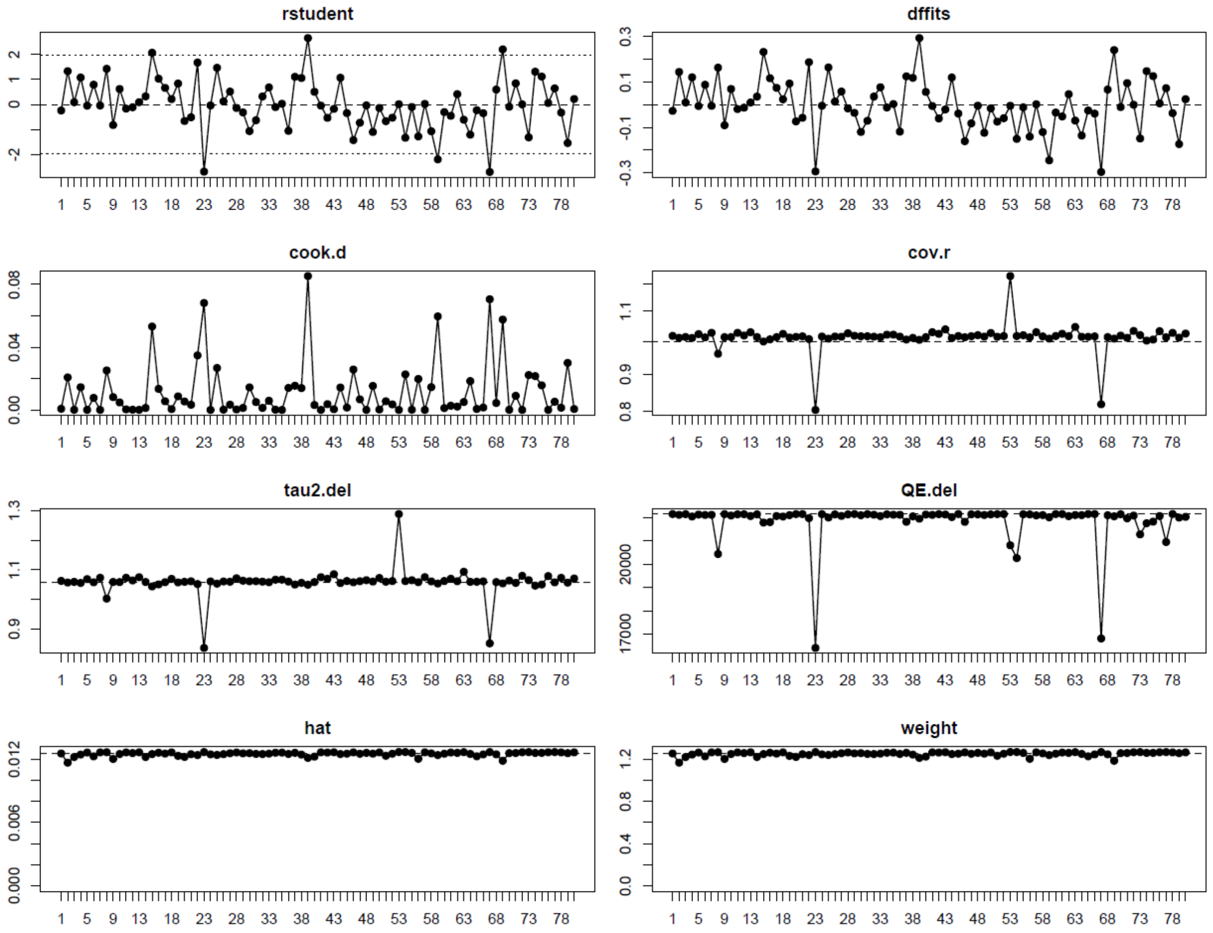

Figure S2. Leave-one-out diagnostics.

The presence of outliers in a meta-analysis may signal a considerable degree of variability within the dataset. This variability could indicate a broad spectrum of responses to the variable or intervention being examined. However, since these outliers did not wield significant influence, their impact on the overall conclusions drawn from the analysis was likely minimal. In this scenario, the robustness of the meta-analysis results was demonstrated by their resilience to these outliers. Therefore, it lends credence to the reliability of the conclusions derived from the analysis. Despite the outliers, the core findings of the meta-analysis remained consistent, underscoring their dependability. This robustness against non-influential outliers also suggested that the overall conclusions, while accounting for a wide range of responses, were not skewed by extreme data points. Consequently, the insights generated from this meta-analysis was considered reliable and indicative of the larger trends within the studied data.

3.3. Random effect model results for the subgroups

In addition to the results from subsection 2.1, the prevalence of TMDs were analyzed in subgroups, namely by continent, age group, gender, and diagnostic instrument.

3.3.1. The prevalence of TMDs by continent

The results of the subgroup analysis by continent are shown in Table S2.

Table S2. Results of random effect model with stratification by continent

Continent	*k*	*Proportion*	*95% CI*	τ^2	τ	*Q*	I^2
Asia	31	0.33	[0.28 – 0.39]	0.53	0.74	4325.39	99.3%

South America	18	0.47	[0.39 – 0.55]	0.47	0.68	1114.61	98.5%
North America	5	0.26	[0.06 – 0.65]	3.54	1.88	1135.53	99.6%
Europe	26	0.29	[0.21 – 0.39]	1.35	1.16	8818.22	99.7%

The heterogeneity analysis of the subgroups reveals some patterns. Asia and South America display moderate between-study variance, as indicated by their τ^2 values. On the other hand, Europe and North America demonstrate considerably higher between-study variance. The Q values for Europe and Asia were particularly high, underscoring the fact that the observed effect sizes within these continents deviate significantly from what could be attributed to sampling error alone. This suggested a substantial degree of variation in the studies within these regions. Moreover, the I^2 values for all continents were strikingly high, nearing 100%. This indicated that almost all the variability in the effect sizes across the studies within each continent stemmed from true heterogeneity, i.e., real differences in effect size, as opposed to chance variations.

A test for subgroup differences, $Q(3) = 10.54$, yields a $p = 0.015$, denoting statistically significant differences between the continents. This suggests that geographical location might play a role in the outcome of the studies. Upon further analysis of confidence intervals, it becomes evident that the prevalence of TMDs was significantly higher in South America compared to Asia and Europe. However, no significant differences were observed among the other groups.

The results by forest plot prevalence of TMDs by continent can be found in Supplementary Material S4.

3.3.2. The prevalence of TMDs by age

The results of the subgroup analysis by age group are shown in Table S3.

Table S3. Results of random effect model with stratification by age

Age	*k*	*Proportion*	*95% CI*	τ^2	τ	*Q*	I^2
Up to 18 yrs.	34	0.27	[0.21 – 0.34]	1.03	1.01	6312.51	99.5%
18-60 yrs.	32	0.41	[0.36 – 0.46]	0.35	0.59	2747.76	98.9%
60+ yrs.	5	0.36	[0.14 – 0.66]	2.04	1.43	1145.23	99.7%

The data in Table S3 suggested that the age group of 18-60 years has the highest proportion of TMDs, followed by the 60+ years group, and then the group up to 18 years. However, due to the large confidence interval in the 60+ years group, it was unclear if the true proportion might actually be higher or lower than the other groups.

The difference in the proportion of TMDs cases among the age groups could be influenced by the high heterogeneity within each group. The highest heterogeneity is observed in the 60+ years group, which could explain the wide confidence interval and the uncertainty around the proportion of TMDs in this age group. While the number of studies might influence the heterogeneity to some extent, it did not appear to be the primary determinant. The relatively lower heterogeneity in the 18-60 years group could contribute to a more consistent and higher proportion of TMDs observed in this group across different studies. For the group up to 18 years, the high heterogeneity might reflect the diversity of factors influencing TMDs in this young population, thus impacting the observed proportion.

A test for subgroup differences, represented as $Q(2) = 9.49$, $p = 0.009$, signifying a statistically significant variation among different age brackets. This finding implied a potential influence of age on the study outcomes. On delving deeper into the confidence intervals, it becomes clear that TMDs were notably more prevalent in patients aged between 18-60 years compared to those aged 18 years and below. This marked difference underscores the potential influence of age on TMDs prevalence.

The results by forest plot prevalence of TMDs by age can be found in Supplementary Material S4.

3.3.3. The prevalence of TMDs by continent and age

Subgroups were considered in isolation by missing some age groups in the study group for South America.

Asia

The results of the subgroup analysis by diagnostic tool are shown in Table S4.

Table S4. Results of random effect model with stratification by Continent (Asia) and age

Age	*k*	*Proportion*	*95% CI*	τ^2	τ
Up to 18 yrs.	12	0.32	[0.24 – 0.41]	0.92	0.96
18-60 yrs.	18	0.35	[0.28 – 0.43]	0.24	0.49
60+ yrs.	1	0.14	[0.04 – 0.39]	-	-

The test for subgroup differences using a random effects model yielded a Q(2) = 2.79 between groups. The p-value for the between-group differences was 0.2483, indicating that the differences in TMDs diagnoses proportions across the age groups were not statistically significant.

The age group 18-60 showed a proportion of TMDs diagnoses that was close to and slightly higher than the overall mean sample proportion. The age group up to 18 also demonstrated a similar trend, but with a slightly lower proportion. In contrast, the age group of 60 and above exhibited a significantly lower proportion of TMDs diagnoses compared to the mean sample proportion.

However, the conclusions drawn from these findings should be interpreted with caution, particularly for the 60+ age group. The proportion for this group was based on a single study, which limits the extent to which these findings can be generalized to the broader population. This limitation underlines the need for additional research that involves multiple studies focusing on the 60+ age group to provide a more reliable and generalizable estimate of TMDs occurrence in this age demographic.

The results by forest plot prevalence of TMDs continent (Asia) and age can be found in Supplementary Material S4.

South America

The results of the subgroup analysis by diagnostic tool are shown in Table S5.

Table S5. Results of random effect model with stratification by Continent (South America) and age

Age	*k*	*Proportion*	*95% CI*	τ^2	τ
Up to 18 yrs.	6	0.33	[0.28 – 0.38]	0.05	0.23
18-60 yrs.	8	0.56	[0.43 – 0.69]	0.58	0.76
60+ yrs.	2	0.56	[0.45 – 0.66]	0.07	0.27

The test for subgroup differences using a random effects model yielded a Q (2) = 22.89, $p < 0.001$. This indicated that the differences in TMDs diagnoses proportions across the age groups were statistically significant. The proportions of TMDs diagnoses in the 18-60 yrs. and 60+ age yrs. groups were significantly higher than in the age group up to 18 yrs.. The 18-60 age group also showed the highest degree of variability across studies. These findings suggest that age may be an important factor influencing the prevalence of TMDs diagnoses, with more variability observed in the middle-age group.

Both the 18-60 yrs. and 60+ yrs. age groups showed a higher proportion of TMDs diagnoses compared to the mean sample proportion. The age group up to 18 had a proportion that was slightly lower than the mean sample proportion.

The results by forest plot prevalence of TMDs continent (South America) and age can be found in Supplementary Material S4.

North America

The results of the subgroup analysis by diagnostic tool are shown in Table S6.

Table S6. Results of random effect model with stratification by Continent (North America) and age

Age	*k*	*Proportion*	*95% CI*	τ^2	τ
Up to 18 yrs.	4	0.37	[0.17 – 0.62]	1.05	
18-60 yrs.	-	-	-	-	-
60+ yrs.	-	-	-	-	-

The proportion of TMDs diagnoses in the "Up to 18 years" subgroup was slightly higher than the mean sample proportion. However, the confidence interval for this age group was wider than that of the mean sample proportion, indicating a high degree of uncertainty around the estimate. This is reflected in the high τ^2 value, which suggested considerable heterogeneity in the results of the individual studies within this age group.

The lower bound of the CI for this group was below the overall mean, while the upper bound was significantly above. This suggests that while on average this group may have a higher proportion of TMDs diagnoses, there is considerable variation in the results.

The results by forest plot prevalence of TMDs continent (North America) and age can be found in Supplementary Material S4.

Europe

The results of the subgroup analysis by diagnostic tool are shown in Table S7.

Table S7. Results of random effect model with stratification by Continent (Europe) and age

Age	*k*	*Proportion*	*95% CI*	τ^2	τ
Up to 18 yrs.	12	0.18	[0.10 – 0.31]	1.42	1.19
18-60 yrs.	6	0.41	[0.32 – 0.51]	0.21	0.46
60+ yrs.	2	0.32	[0.04 – 0.84]	3.07	1.75

Comparing these to the mean sample proportion (0.34 with a 95% CI [0.29, 0.39]), we note: the "up to 18" age group had a lower TMDs diagnoses proportion than the mean; the "18-60" age group had a higher TMDs diagnoses proportion than the mean; the "60+" age group, despite its lower TMDs diagnoses proportion, exhibited substantial heterogeneity.

The test for subgroup differences using a random effects model yielded a $Q(2) = 8.31$ with a p-value of 0.016. This indicated that the differences in TMDs diagnoses proportions across the age groups were statistically significant. Based on the confidence intervals, there was a significantly lower proportion of TMDs in the up to 18-yrs. compared with 18-60 yrs.

The high τ^2 values, especially for the "Age up to 18" and "Age 60+" groups, suggested a high level of variability between studies within the same age groups. This could be due to methodological differences between studies or variations in the populations studied.

The results by forest plot prevalence of TMDs continent (Europe) and age can be found in Supplementary Material S4.

3.3.4. Distribution of the female-to-male ratio

The distribution of the female-to-male ratio (F:M) across different continents was studied, with the results outlined in Table S8.

Table S8. The distribution of the female-to-male ratio (F:M) across different continents.

	N	*Asia,* *n = 31*[1]	*Europe,* *n = 26*[1]	*North America,* *n = 5*[1]	*South America,* *n = 18*[1]	*p*[2]
F:M	54	1.26 (1.04, 1.84)	1.09 (1.00, 1.46)	1.26 (1.16, 1.34)	1.56 (1.25, 2.20)	0.286
Missing values		11	9	1	5	

[1] Median (Q1, Q3)

[2] Kruskal-Wallis rank sum test

From the data presented, we observed that for each continent, the size of the female group was larger than the male group by an average of 9% to 56%. The highest F:M was reported in South America (1.56), conversely, Europe reported the lowest F:M (1.09), suggesting a near-equal distribution of males and females. However, the Kruskal-Wallis test, a non-parametric method used to compare two or more independent samples of equal or different sample sizes, yielded a p-value of 0.286. This value was greater than 0.05, suggesting that the differences in F:M across continents were not statistically significant. In conclusion, while regional variations in TMDs diagnoses by gender did exist, they may not have been large enough to be considered statistically significant.

3.4. Assessment of publication bayes

The funnel plot for N =80 studies is shown in Figure S3.

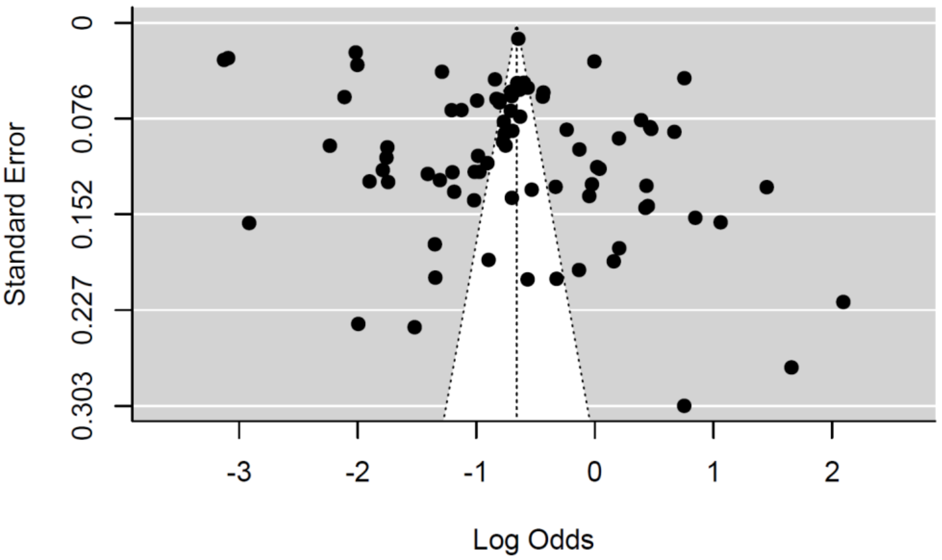

Figure S3. The funnel plot (points stand for studies)

A funnel plot, based on a random-effects model, was constructed to visually inspect potential publication bias. The estimated log odds of -0.66, derived from our model, was indicated by a vertical line. A pseudo confidence interval region, calculated as ± 1.96 times the standard error, was drawn around this estimate. Upon visual inspection, the funnel plot exhibited a largely symmetrical distribution of points about the X-axis on either side of this centerline. Along the Y-axis, we observed a higher concentration of points near the apex, indicative of smaller standard errors for the majority of studies.

To statistically confirm the observed symmetry of the funnel plot, we applied the trim and fill method. This method estimates the number of missing studies on the left side of the funnel plot, which typically represent studies with smaller sample sizes or more negative effect sizes. In our analysis, the estimated number of missing studies by trim and fill was zero, suggesting no evidence of publication bias. This was visually corroborated by the fact that the imputed funnel plot was identical to the original one.

Next, we conducted the Egger's test, which resulted in $t(78) = 1.96$, $p = 0.053$. This borderline significant p-value suggests that the slight asymmetry observed in the funnel plot could be due to chance. However, the negative limit estimate (-1.30) with a 95% confidence interval of [-1.64, -0.96] indicated a trend for smaller studies (those with larger standard errors) to report more negative effect sizes.

Finally, we performed the rank correlation test, which yielded $\tau_{rc} = -0.04$, $p = 0.646$. This non-significant result indicated no substantial evidence that smaller studies with larger standard errors are reporting more extreme effects. In other words, we found no significant evidence of publication bias.
In conclusion, both visual and statistical assessments suggest that our meta-analysis results were robust and unlikely to be significantly affected by publication bias.

References

1. Dworkin, S.F.; LeResche, L. Research Diagnostic Criteria for Temporomandibular Disorders: Review, Criteria, Examinations and Specifications, Critique. *J. Craniomandib. Disord. Facial Oral Pain* **1992**, *6*, 301–355.
2. Fonseca, D.M. da; Bonfante, G.; Valle, A.L. do; Freitas, S.F.T. de Diagnóstico pela anamnese da disfunção craniomandibular. *RGO Porto Alegre* **1994**, 23–28.
3. Booth, A. Over 85% of Included Studies in Systematic Reviews Are on MEDLINE. *J. Clin. Epidemiol.* **2016**, *79*, 165–166, doi:10.1016/j.jclinepi.2016.04.002.
4. Halladay, C.W.; Trikalinos, T.A.; Schmid, I.T.; Schmid, C.H.; Dahabreh, I.J. Using Data Sources beyond PubMed Has a Modest Impact on the Results of Systematic Reviews of Therapeutic Interventions. *J. Clin. Epidemiol.* **2015**, *68*, 1076–1084, doi:10.1016/j.jclinepi.2014.12.017.
5. Holden, B.A.; Fricke, T.R.; Wilson, D.A.; Jong, M.; Naidoo, K.S.; Sankaridurg, P.; Wong, T.Y.; Naduvilath, T.J.; Resnikoff, S. Global Prevalence of Myopia and High Myopia and Temporal Trends from 2000 through 2050. *Ophthalmology* **2016**, *123*, 1036–1042, doi:10.1016/j.ophtha.2016.01.006.
6. Rice, D.B.; Kloda, L.A.; Levis, B.; Qi, B.; Kingsland, E.; Thombs, B.D. Are MEDLINE Searches Sufficient for Systematic Reviews and Meta-Analyses of the Diagnostic Accuracy of Depression Screening Tools? A Review of Meta-Analyses. *J. Psychosom. Res.* **2016**, *87*, 7–13, doi:10.1016/j.jpsychores.2016.06.002.
7. Valesan, L.F.; Da-Cas, C.D.; Réus, J.C.; Denardin, A.C.S.; Garanhani, R.R.; Bonotto, D.; Januzzi, E.; de Souza, B.D.M. Prevalence of Temporomandibular Joint Disorders: A Systematic Review and Meta-Analysis. *Clin. Oral Investig.* **2021**, *25*, 441–453, doi:10.1007/s00784-020-03710-w.
8. Khan, K.; Muller-Bolla, M.; Anacleto Teixeira Junior, O.; Gornitsky, M.; Guimarães, A.S.; Velly, A.M. Comorbid Conditions Associated with Painful Temporomandibular Disorders in Adolescents from Brazil, Canada and France: A Cross-Sectional Study. *J. Oral Rehabil.* **2020**, *47*, 417–424, doi:10.1111/joor.12923.
9. Fonseca, F.F.; Politti, F.; Cunha, T.; Leonardis, M.; Carvalho, L.; de Paula Gomes, C.A.F.; Biasotto-Gonzalez, D.A. Prevalence of Signs and Symptoms of Temporomandibular Disorder in the Metropolitan Region of Rio De Janeiro: A Population-Based Cross-Sectional Study. *CRANIO®* **2022**, *0*, 1–7, doi:10.1080/08869634.2022.2091099.
10. Oliveira, A.S. de; Dias, E.M.; Contato, R.G.; Berzin, F. Prevalence Study of Signs and Symptoms of Temporomandibular Disorder in Brazilian College Students. *Braz. Oral Res.* **2006**, *20*, 3–7, doi:10.1590/S1806-83242006000100002.
11. Pedroni, C.R.; De Oliveira, A.S.; Guaratini, M.I. Prevalence Study of Signs and Symptoms of Temporomandibular Disorders in University Students. *J. Oral Rehabil.* **2003**, *30*, 283–289, doi:10.1046/j.1365-2842.2003.01010.x.
12. Fernandes Azevedo, A.B.; Câmara-Souza, M.B.; Dantas, I. de S.; de Resende, C.M.B.M.; Barbosa, G.A.S. Relationship between Anxiety and Temporomandibular Disorders in Dental Students. *CRANIO®* **2018**, *36*, 300–303, doi:10.1080/08869634.2017.1361053.
13. Camacho, J.G.D.D.; Oltramari-Navarro, P.V.P.; Navarro, R. de L.; Conti, A.C. de C.F.; Conti, M.R. de A.; Marchiori, L.L. de M.; Fernandes, K.B.P. Signs and Symptoms of Temporomandibular Disorders in the Elderly. *CoDAS* **2014**, *26*, 76–80, doi:10.1590/S2317-17822014000100011.
14. Gonçalves, D.A. de G.; Dal Fabbro, A.L.; Campos, J.A.D.B.; Bigal, M.E.; Speciali, J.G. Symptoms of Temporomandibular Disorders in the Population: An Epidemiological Study. *J. Orofac. Pain* **2010**, *24*, 270–278.
15. Campos, J.A.D.B.; Carrascosa, A.C.; Bonafé, F.S.S.; Maroco, J. Epidemiology of Severity of Temporomandibular Disorders in Brazilian Women. *J. Oral Facial Pain Headache* **2014**, *28*, 147–152, doi:10.11607/ofph.1194.

16. Sampaio, N. de M.; Oliveira, M.C.; Ortega, A. de O.; Santos, L. de B.; Alves, T.D.B. Temporomandibular Disorders in Elderly Individuals: The Influence of Institutionalization and Sociodemographic Factors. *CoDAS* **2017**, *29*, e20160114, doi:10.1590/2317-1782/20162016114.
17. Figueiredo Ribeiro, D.C.; Ferreira Gradella, C.M.; Franco Rocha Rodrigues, L.L.; Abanto, J.; Oliveira, L.B. The Impact of Temporomandibular Disorders on the Oral Health-Related Quality of Life of Brazilian Children: A Cross-Sectional Study. *J. Dent. Child.* **2020**, *87*, 103–109.
18. Dallanora, A.F.; Grasel, C.E.; Heine, C.P.; Demarco, F.F.; Pereira-Cenci, T.; Presta, A.A.; Boscato, N. Prevalence of Temporomandibular Disorders in a Population of Complete Denture Wearers. *Gerodontology* **2012**, *29*, e865–e869, doi:10.1111/j.1741-2358.2011.00574.x.
19. Ramírez-Caro, S.N.; Espinosa de Santillana, I.A.; Muñoz-Quintana, G. [Prevalence of temporomandibular disorders in Mexican children with mixed dentition]. *Rev. Salud Publica Bogota Colomb.* **2015**, *17*, 289–299, doi:10.15446/rsap.v17n2.27958.
20. Mendiburu-Zavala, C.E.; Castillero-Rosas, A.S.; Lugo-Ancona, P.E.; Carrillo-Mendiburu, J. Disfunción Temporomandibular y Depresión En Adolescentes de Ascendencia Maya. *Bol. Méd. Hosp. Infant. México* **2020**, *77*, doi:10.24875/BMHIM.20000002.
21. Karthik, R.; Hafila, M.I.F.; Saravanan, C.; Vivek, N.; Priyadarsini, P.; Ashwath, B. Assessing Prevalence of Temporomandibular Disorders among University Students: A Questionnaire Study. *J. Int. Soc. Prev. Community Dent.* **2017**, *7*, S24–S29, doi:10.4103/jispcd.JISPCD_146_17.
22. Bahrani, F.; Ghadiri, P.; Vojdani, M. Comparison of Temporomandibular Disorders in Iranian Dental and Nondental Students. *J. Contemp. Dent. Pract.* **2012**, *13*, 173–177, doi:10.5005/jp-journals-10024-1116.
23. Srivastava, K.C.; Shrivastava, D.; Khan, Z.A.; Nagarajappa, A.K.; Mousa, M.A.; Hamza, M.O.; Al-Johani, K.; Alam, M.K. Evaluation of Temporomandibular Disorders among Dental Students of Saudi Arabia Using Diagnostic Criteria for Temporomandibular Disorders (DC/TMD): A Cross-Sectional Study. *BMC Oral Health* **2021**, *21*, 211, doi:10.1186/s12903-021-01578-0.
24. Alketbi, N.; Talaat, W. Prevalence and Characteristics of Referred Pain in Patients Diagnosed with Temporomandibular Disorders According to the Diagnostic Criteria for Temporomandibular Disorders (DC/TMD) in Sharjah, United Arab Emirates 2022.
25. Habib, S.R.; Al Rifaiy, M.Q.; Awan, K.H.; Alsaif, A.; Alshalan, A.; Altokais, Y. Prevalence and Severity of Temporomandibular Disorders among University Students in Riyadh. *Saudi Dent. J.* **2015**, *27*, 125–130, doi:10.1016/j.sdentj.2014.11.009.
26. Marpaung, C.; van Selms, M.K.A.; Lobbezoo, F. Prevalence and Risk Indicators of Pain-Related Temporomandibular Disorders among Indonesian Children and Adolescents. *Community Dent. Oral Epidemiol.* **2018**, *46*, 400–406, doi:10.1111/cdoe.12382.
27. Nekora-Azak, A.; Evlioglu, G.; Ordulu, M.; İşsever, H. Prevalence of Symptoms Associated with Temporomandibular Disorders in a Turkish Population. *J. Oral Rehabil.* **2006**, *33*, 81–84, doi:10.1111/j.1365-2842.2006.01543.x.
28. Jomhawi, J.M.; Elsamarneh, A.M.; Hassan, A.M. Prevalence of Temporomandibular Disorder among Schoolchildren in Jordan. *Int. J. Clin. Pediatr. Dent.* **2021**, *14*, 304–310, doi:10.5005/jp-journals-10005-1939.
29. Alkhubaizi, Q.; Khalaf, M.E.; Faridoun, A. Prevalence of Temporomandibular Disorder-Related Pain among Adults Seeking Dental Care: A Cross-Sectional Study. *Int. J. Dent.* **2022**, *2022*, e3186069, doi:10.1155/2022/3186069.
30. Talaat, W.M.; Adel, O.I.; Al Bayatti, S. Prevalence of Temporomandibular Disorders Discovered Incidentally during Routine Dental Examination Using the Research Diagnostic Criteria for Temporomandibular Disorders. *Oral Surg. Oral Med. Oral Pathol. Oral Radiol.* **2018**, *125*, 250–259, doi:10.1016/j.oooo.2017.11.012.
31. Kmeid, E.; Nacouzi, M.; Hallit, S.; Rohayem, Z. Prevalence of Temporomandibular Joint Disorder in the Lebanese Population, and Its Association with Depression, Anxiety, and Stress. *Head Face Med.* **2020**, *16*, 19, doi:10.1186/s13005-020-00234-2.

32. Feteih, R.M. Signs and Symptoms of Temporomandibular Disorders and Oral Parafunctions in Urban Saudi Arabian Adolescents: A Research Report. *Head Face Med.* **2006**, *2*, 25, doi:10.1186/1746-160X-2-25.
33. Natu, V.P.; Yap, A.U.-J.; Su, M.H.; Irfan Ali, N.M.; Ansari, A. Temporomandibular Disorder Symptoms and Their Association with Quality of Life, Emotional States and Sleep Quality in South-East Asian Youths. *J. Oral Rehabil.* **2018**, *45*, 756–763, doi:10.1111/joor.12692.
34. Taneja, P.; Nagpal, R.; Marya, C.M.; Kataria, S.; Sahay, V.; Goyal, D. Temporomandibular Disorders among Adolescents of Haryana, India: A Cross-Sectional Study. *Int. J. Clin. Pediatr. Dent.* **2019**, *12*, 500–506, doi:10.5005/jp-journals-10005-1689.
35. Wu, J.; Huang, Z.; Chen, Y.; Chen, Y.; Pan, Z.; Gu, Y. Temporomandibular Disorders among Medical Students in China: Prevalence, Biological and Psychological Risk Factors. *BMC Oral Health* **2021**, *21*, 549, doi:10.1186/s12903-021-01916-2.
36. Ebrahimi, M.; Dashti, H.; Mehrabkhani, M.; Arghavani, M.; Daneshvar-Mozafari, A. Temporomandibular Disorders and Related Factors in a Group of Iranian Adolescents: A Cross-Sectional Survey. *J. Dent. Res. Dent. Clin. Dent. Prospects* **2011**, *5*, 123–127, doi:10.5681/joddd.2011.028.
37. Choi, Y.-S.; Choung, P.-H.; Moon, H.-S.; Kim, S.-G. Temporomandibular Disorders in 19-Year-Old Korean Men. *J. Oral Maxillofac. Surg.* **2002**, *60*, 797–803, doi:10.1053/joms.2002.33249.
38. Lei, J.; Fu, J.; Yap, A.U.J.; Fu, K.-Y. Temporomandibular Disorders Symptoms in Asian Adolescents and Their Association with Sleep Quality and Psychological Distress. *CRANIO®* **2016**, *34*, 242–249, doi:10.1179/2151090315Y.0000000021.
39. Özdinç, S.; Ata, H.; Selçuk, H.; Can, H.B.; Sermenli, N.; Turan, F.N. Temporomandibular Joint Disorder Determined by Fonseca Anamnestic Index and Associated Factors in 18- to 27-Year-Old University Students. *CRANIO®* **2020**, *38*, 327–332, doi:10.1080/08869634.2018.1513442.
40. Yasuda, E.; Honda, K.; Hasegawa, Y.; Matsumura, E.; Fujiwara, M.; Hasegawa, M.; Kishimoto, H. Prevalence of Temporomandibular Disorders among Junior High School Students Who Play Wind Instruments. *Int. J. Occup. Med. Environ. Health* **2015**, *29*, 69–76, doi:10.13075/ijomeh.1896.00524.
41. Prakash, J.; Ranvijay, K.; Devi, L.S.; Shenoy, M.; Abdul, N.S.; Shivakumar, G.C.; Gupta, P. Assessment of Symptoms Associated with Temporomandibular Dysfunction and Bruxism among Elderly Population: An Epidemiological Survey. *J. Contemp. Dent. Pract.* **2022**, *23*, 393–398.
42. Nourallah, H.; Johansson, A. Prevalence of Signs and Symptoms of Temporomandibular Disorders in a Young Male Saudi Population. *J. Oral Rehabil.* **1995**, *22*, 343–347, doi:10.1111/j.1365-2842.1995.tb00783.x.
43. Pow, E.H.; Leung, K.C.; McMillan, A.S. Prevalence of Symptoms Associated with Temporomandibular Disorders in Hong Kong Chinese. *J. Orofac. Pain* **2001**, *15*, 228–234.
44. Verdonck, A.; Takada, K.; Kitai, N.; Kuriama, R.; Yasuda, Y.; Carels, C.; Sakuda, M. The Prevalence of Cardinal TMJ Dysfunction Symptoms and Its Relationship to Occlusal Factors in Japanese Female Adolescents. *J. Oral Rehabil.* **1994**, *21*, 687–697, doi:10.1111/j.1365-2842.1994.tb01184.x.
45. Akhter, R.; Hassan, N.M.M.; Ohkubo, R.; Tsukazaki, T.; Aida, J.; Morita, M. The Relationship between Jaw Injury, Third Molar Removal, and Orthodontic Treatment and TMD Symptoms in University Students in Japan. *J. Orofac. Pain* **2008**, *22*, 50–56.
46. Barbosa, C.; Manso, M.C.; Reis, T.; Soares, T.; Gavinha, S.; Ohrbach, R. Are Oral Overuse Behaviours Associated with Painful Temporomandibular Disorders? A Cross-Sectional Study in Portuguese University Students. *J. Oral Rehabil.* **2021**, *48*, 1099–1108, doi:10.1111/joor.13226.
47. Graue, A.M.; Jokstad, A.; Assmus, J.; Skeie, M.S. Prevalence among Adolescents in Bergen, Western Norway, of Temporomandibular Disorders According to the DC/TMD Criteria and Examination Protocol. *Acta Odontol. Scand.* **2016**, *74*, 449–455, doi:10.1080/00016357.2016.1191086.

48. Perrotta, S.; Bucci, R.; Simeon, V.; Martina, S.; Michelotti, A.; Valletta, R. Prevalence of Malocclusion, Oral Parafunctions and Temporomandibular Disorder-Pain in Italian Schoolchildren: An Epidemiological Study. *J. Oral Rehabil.* **2019**, *46*, 611–616, doi:10.1111/joor.12794.
49. Marpaung, C.; Lobbezoo, F.; van Selms, M.K.A. Temporomandibular Disorders among Dutch Adolescents: Prevalence and Biological, Psychological, and Social Risk Indicators. *Pain Res. Manag.* **2018**, *2018*, e5053709, doi:10.1155/2018/5053709.
50. Hadler-Olsen, E.; Thon, E.; Holde, G.E.; Jönsson, B.; Oscarson, N.; Tillberg, A. Temporomandibular Disorders in an Adult Population in Northern Norway: A Cross-Sectional Study. *Clin. Exp. Dent. Res.* **2021**, *7*, 1144–1153, doi:10.1002/cre2.463.
51. Storm, C.; Wänman, A. Temporomandibular Disorders, Headaches, and Cervical Pain among Females in a Sami Population. *Acta Odontol. Scand.* **2006**, *64*, 319–325, doi:10.1080/00016350600801915.
52. Loster, J.E.; Osiewicz, M.A.; Groch, M.; Ryniewicz, W.; Wieczorek, A. The Prevalence of TMD in Polish Young Adults. *J. Prosthodont.* **2017**, *26*, 284–288, doi:10.1111/jopr.12414.
53. Ciancaglini, R.; Radaelli, G. The Relationship between Headache and Symptoms of Temporomandibular Disorder in the General Population. *J. Dent.* **2001**, *29*, 93–98, doi:10.1016/S0300-5712(00)00042-7.
54. Vainionpää, R.; Kinnunen, T.; Pesonen, P.; Laitala, M.-L.; Anttonen, V.; Sipilä, K. Prevalence of Temporomandibular Disorders (TMD) among Finnish Prisoners: Cross-Sectional Clinical Study. *Acta Odontol. Scand.* **2019**, *77*, 264–268, doi:10.1080/00016357.2018.1535660.
55. Gesch, D.; Bernhardt, O.; Alte, D.; Schwahn, C.; Kocher, T.; John, U.; Hensel, E. Prevalence of Signs and Symptoms of Temporomandibular Disorders in an Urban and Rural German Population: Results of a Population-Based Study of Health in Pomerania. *Quintessence Int. Berl. Ger. 1985* **2004**, *35*, 143–150.
56. Nilsson, I.-M.; List, T.; Drangsholt, M. Prevalence of Temporomandibular Pain and Subsequent Dental Treatment in Swedish Adolescents. *J. Orofac. Pain* **2005**, *19*, 144–150.
57. Rantala, M.A.; Ahlberg, J.; Suvinen, T.I.; Savolainen, A.; Könönen, M. Symptoms, Signs, and Clinical Diagnoses According to the Research Diagnostic Criteria for Temporomandibular Disorders among Finnish Multiprofessional Media Personnel. *J. Orofac. Pain* **2003**, *17*, 311–316.
58. Banafa, A.; Suominen, A.L.; Sipilä, K. Factors Associated with Signs of Temporomandibular Pain: An 11-Year-Follow-up Study on Finnish Adults. *Acta Odontol. Scand.* **2020**, *78*, 57–63, doi:10.1080/00016357.2019.1650955.
59. Carlsson, G.E.; Ekbäck, G.; Johansson, A.; Ordell, S.; Unell, L. Is There a Trend of Decreasing Prevalence of TMD-Related Symptoms with Ageing among the Elderly? *Acta Odontol. Scand.* **2014**, *72*, 714–720, doi:10.3109/00016357.2014.898787.
60. Yekkalam, N.; Wänman, A. Prevalence of Signs and Symptoms Indicative of Temporomandibular Disorders and Headaches in 35-, 50-, 65- and 75-Year-Olds Living in Västerbotten, Sweden. *Acta Odontol. Scand.* **2014**, *72*, 458–465, doi:10.3109/00016357.2013.860620.
61. Adegbiji, W.A.; Olajide, G.T.; Agbesanwa, A.T.; Banjo, O.O. Otological Manifestation of Temporomandibular Joint Disorder in Ekiti, a Sub-Saharan African Country. *J. Int. Med. Res.* **2021**, *49*, 300060521996517, doi:10.1177/0300060521996517.
62. Zwiri, A.M.A.; Al-Omiri, M.K. Prevalence of Temporomandibular Joint Disorder among North Saudi University Students. *CRANIO®* **2016**, *34*, 176–181, doi:10.1179/2151090315Y.0000000007.
63. Bertoli, F.M. de P.; Bruzamolin, C.D.; Pizzatto, E.; Losso, E.M.; Brancher, J.A.; Souza, J.F. de Prevalence of Diagnosed Temporomandibular Disorders: A Cross-Sectional Study in Brazilian Adolescents. *PLOS ONE* **2018**, *13*, e0192254, doi:10.1371/journal.pone.0192254.
64. Medeiros, R.A.D.; Vieira, D.L.; Silva, E.V.F.D.; Rezende, L.V.M.D.L.; Santos, R.W.D.; Tabata, L.F. Prevalence of Symptoms of Temporomandibular Disorders, Oral Behaviors, Anxiety, and Depression in Dentistry Students during the Period of Social Isolation Due to COVID-19. *J. Appl. Oral Sci. 28*, e20200445, doi:10.1590/1678-7757-2020-0445.

65. Júnior, P.C. de M.; Aroucha, J.M.C.N.L.; Arnaud, M.; Lima, M.G. de S.; Gomes, S.G.F.; Ximenes, R.; Rosenblatt, A.; Jr, A. de F.C. Prevalence of TMD and Level of Chronic Pain in a Group of Brazilian Adolescents. *PLOS ONE* **2019**, *14*, e0205874, doi:10.1371/journal.pone.0205874.
66. Progiante, P.S.; Pattussi, M.P.; Lawrence, H.P.; Goya, S.; Grossi, P.K.; Grossi, M.L. Prevalence of Temporomandibular Disorders in an Adult Brazilian Community Population Using the Research Diagnostic Criteria (Axes I and II) for Temporomandibular Disorders (The Maringá Study). *Int. J. Prosthodont.* **2015**, *28*, 600–609, doi:10.11607/ijp.4026.
67. Mello, V.V.C. de; Barbosa, A.C. da S.; Morais, M.P.L. de A.; Gomes, S.G.F.; Vasconcelos, M.M.V.B.; Caldas Júnior, A. de F. Temporomandibular Disorders in a Sample Population of the Brazilian Northeast. *Braz. Dent. J.* **2014**, *25*, 442–446, doi:10.1590/0103-6440201302250.
68. De Stefano, A.A.; Guercio-Mónaco, E.; Uzcátegui, A.; Boboc, A.M.; Barbato, E.; Galluccio, G. Temporomandibular Disorders in Venezuelan and Italian Adolescents. *CRANIO®* **2022**, *40*, 517–523, doi:10.1080/08869634.2020.1801013.
69. Franco-Micheloni, A.L.; Fernandes, G.; de Godoi Gonçalves, D.A.; Camparis, C.M. Temporomandibular Disorders in a Young Adolescent Brazilian Population: Epidemiologic Characterization and Associated Factors. *J. Oral Facial Pain Headache* **2015**, *29*, 242–249, doi:10.11607/ofph.1262.
70. Isong, U.; Gansky, S.A.; Plesh, O. Temporomandibular Joint and Muscle Disorder-Type Pain in US Adults: The National Health Interview Survey. *J. Orofac. Pain* **2008**, *22*, 317–322.
71. Moyaho-Bernal, A.; Lara-Muñoz, M.D.C.; Espinosa-De Santillana, I.; Etchegoyen, G. Prevalence of Signs and Symptoms of Temporomandibular Disorders in Children in the State of Puebla, Mexico, Evaluated with the Research Diagnostic Criteria for Temporomandibular Disorders (RDC/TMD). *Acta Odontol. Latinoam. AOL* **2010**, *23*, 228–233.
72. Song, H.-S.; Shin, J.-S.; Lee, J.; Lee, Y.J.; Kim, M.; Cho, J.-H.; Kim, K.-W.; Park, Y.; Song, H.J.; Park, S.-Y.; et al. Association between Temporomandibular Disorders, Chronic Diseases, and Ophthalmologic and Otolaryngologic Disorders in Korean Adults: A Cross-Sectional Study. *PLOS ONE* **2018**, *13*, e0191336, doi:10.1371/journal.pone.0191336.
73. Alrashdan, M.S.; Nuseir, A.; AL-Omiri, M.K. Prevalence and Correlations of Temporomandibular Disorders in Northern Jordan Using Diagnostic Criteria Axis I. *J. Investig. Clin. Dent.* **2019**, *10*, e12390, doi:10.1111/jicd.12390.
74. Al-Khotani, A.; Naimi-Akbar, A.; Albadawi, E.; Ernberg, M.; Hedenberg-Magnusson, B.; Christidis, N. Prevalence of Diagnosed Temporomandibular Disorders among Saudi Arabian Children and Adolescents. *J. Headache Pain* **2016**, *17*, 41, doi:10.1186/s10194-016-0642-9.
75. Hongxing, L.; Astrøm, A.N.; List, T.; Nilsson, I.-M.; Johansson, A. Prevalence of Temporomandibular Disorder Pain in Chinese Adolescents Compared to an Age-Matched Swedish Population. *J. Oral Rehabil.* **2016**, *43*, 241–248, doi:10.1111/joor.12366.
76. Wu, N.; Hirsch, C. Temporomandibular Disorders in German and Chinese Adolescents. *J. Orofac. Orthop. Fortschritte Kieferorthopädie* **2010**, *71*, 187–198, doi:10.1007/s00056-010-1004-x.
77. Wieckiewicz, M.; Grychowska, N.; Nahajowski, M.; Hnitecka, S.; Kempiak, K.; Charemska, K.; Balicz, A.; Chirkowska, A.; Zietek, M.; Winocur, E. Prevalence and Overlaps of Headaches and Pain-Related Temporomandibular Disorders Among the Polish Urban Population. *J. Oral Facial Pain Headache* **2020**, *34*, 31–39, doi:10.11607/ofph.2386.
78. Qvintus, V.; Sipilä, K.; Le Bell, Y.; Suominen, A.L. Prevalence of Clinical Signs and Pain Symptoms of Temporomandibular Disorders and Associated Factors in Adult Finns. *Acta Odontol. Scand.* **2020**, *78*, 515–521, doi:10.1080/00016357.2020.1746395.
79. Paduano, S.; Bucci, R.; Rongo, R.; Silva, R.; Michelotti, A. Prevalence of Temporomandibular Disorders and Oral Parafunctions in Adolescents from Public Schools in Southern Italy. *CRANIO®* **2020**, *38*, 370–375, doi:10.1080/08869634.2018.1556893.
80. Tecco, S.; Crincoli, V.; Di Bisceglie, B.; Saccucci, M.; Macrí, M.; Polimeni, A.; Festa, F. Signs and Symptoms of Temporomandibular Joint Disorders in Caucasian Children and Adolescents. *CRANIO®* **2011**, *29*, 71–79, doi:10.1179/crn.2011.010.

81. Tecco, S.; Nota, A.; Caruso, S.; Primozic, J.; Marzo, G.; Baldini, A.; Gherlone, E.F. Temporomandibular Clinical Exploration in Italian Adolescents. *CRANIO®* **2019**, *37*, 77–84, doi:10.1080/08869634.2017.1391963.
82. Jussila, P.; Kiviahde, H.; Näpänkangas, R.; Päkkilä, J.; Pesonen, P.; Sipilä, K.; Pirttiniemi, P.; Raustia, A. Prevalence of Temporomandibular Disorders in the Northern Finland Birth Cohort 1966. *J. Oral Facial Pain Headache* **2017**, *31*, 159–164, doi:10.11607/ofph.1773.
83. Yu, Q.; Liu, Y.; Chen, X.; Chen, D.; Xie, X.; Hong, X.; Wang, X.; Hung, H.; Yu, Y. Prevalence and Associated Factors for Temporomandibular Disorders in Chinese Civilian Pilots. *Int. Arch. Occup. Environ. Health* **2015**, *88*, doi:10.1007/s00420-015-1018-1.
84. Friedman Rubin, P.; Erez, A.; Peretz, B.; Birenboim-Wilensky, R.; Winocur, E. Prevalence of Bruxism and Temporomandibular Disorders among Orphans in Southeast Uganda: A Gender and Age Comparison. *CRANIO®* **2018**, *36*, 243–249, doi:10.1080/08869634.2017.1331784.
85. Lung, J.; Bell, L.; Heslop, M.; Cuming, S.; Ariyawardana, A. Prevalence of Temporomandibular Disorders among a Cohort of University Undergraduates in Australia. *J. Investig. Clin. Dent.* **2018**, *9*, e12341, doi:10.1111/jicd.12341.
86. Viechtbauer, W.; Cheung, M.W.-L. Outlier and Influence Diagnostics for Meta-Analysis. *Res. Synth. Methods* **2010**, *1*, 112–125, doi:10.1002/jrsm.11.
87. Guarda-Nardini, L.; Piccotti, F.; Mogno, G.; Favero, L.; Manfredini, D. Age-Related Differences in Temporomandibular Disorder Diagnoses. *Cranio J. Craniomandib. Pract.* **2012**, *30*, 103–109, doi:10.1179/crn.2012.015.
88. DerSimonian, R.; Laird, N. Meta-Analysis in Clinical Trials. *Control. Clin. Trials* **1986**, *7*, 177–188, doi:10.1016/0197-2456(86)90046-2.
89. Raudenbush, S.W. Analyzing Effect Sizes: Random-Effects Models. In *The handbook of research synthesis and meta-analysis, 2nd ed*; Russell Sage Foundation: New York, NY, US, 2009; pp. 295–315 ISBN 978-0-87154-163-5.
90. Jackson, D. Confidence Intervals for the Between-Study Variance in Random Effects Meta-Analysis Using Generalised Cochran Heterogeneity Statistics. *Res. Synth. Methods* **2013**, *4*, 220–229, doi:10.1002/jrsm.1081.
91. Higgins, J.P.T.; Thompson, S.G. Quantifying Heterogeneity in a Meta-analysis. *Stat. Med.* **2002**, *21*, 1539–1558, doi:10.1002/sim.1186.
92. Duval, S.; Tweedie, R. Trim and Fill: A Simple Funnel-Plot–Based Method of Testing and Adjusting for Publication Bias in Meta-Analysis. *Biometrics* **2000**, *56*, 455–463, doi:10.1111/j.0006-341X.2000.00455.x.
93. Duval, S.; Tweedie, R. A Nonparametric "Trim and Fill" Method of Accounting for Publication Bias in Meta-Analysis. *J. Am. Stat. Assoc.* **2000**, *95*, 89–98, doi:10.1080/01621459.2000.10473905.
94. Egger, M.; Smith, G.D.; Schneider, M.; Minder, C. Bias in Meta-Analysis Detected by a Simple, Graphical Test. *BMJ* **1997**, *315*, 629–634, doi:10.1136/bmj.315.7109.629.
95. Begg, C.B.; Mazumdar, M. Operating Characteristics of a Rank Correlation Test for Publication Bias. *Biometrics* **1994**, *50*, 1088, doi:10.2307/2533446.
96. Balduzzi, S.; Rücker, G.; Schwarzer, G. How to Perform a Meta-Analysis with R: A Practical Tutorial. *Evid. Based Ment. Health* **2019**, *22*, 153–160, doi:10.1136/ebmental-2019-300117.
97. Coburn, K.; Vevea, J. Weightr: Estimating Weight-Function Models for Publication Bias. R Package Version 2.0.2 Available online: https://CRAN.R-project.org/package=weightr.
98. Makowski, D.; Lüdecke, D.; Patil, I.; Thériault, R.; Ben-Shachar, M.; Wiernik, B. Automated Results Reporting as a Practical Tool to Improve Reproducibility and Methodological Best Practices Adoption. Available online: URL: https://easystats.github.io/report/ (accessed on 16 November 2023).
99. Schwarzer, G.; Carpenter, J.R.; Rücker, G. Metasens: Statistical Methods for Sensitivity Analysis in Meta-Analysis 2023.
100. Viechtbauer, W. Conducting Meta-Analyses in R with the Metafor Package. *J. Stat. Softw.* **2010**, *36*, doi:10.18637/jss.v036.i03.

101. Wickham, H. *Ggplot2: Elegant Graphics for Data Analysis*; Use R!; 2nd ed. 2016.; Springer International Publishing : Imprint: Springer: Cham, 2016; ISBN 978-3-319-24277-4.
102. Wickham, H.; Bryan, J.; Posit; attribution), P. (Copyright holder of all R. code and all C. code without explicit copyright; code), M.K. (Author of included R.; code), K.V. (Author of included libxls; code), C.L. (Author of included libxls; code), B.C. (Author of included libxls; code), D.H. (Author of included libxls; code), E.M. (Author of included libxls Readxl: Read Excel Files 2023.
103. Wickham, H.; François, R.; Henry, L.; Müller, K.; Vaughan, D.; Software, P.; PBC Dplyr: A Grammar of Data Manipulation 2023.
104. Harrer, M.; Cuijpers, P.; Furukawa, T.; Ebert, D. Dmetar: Companion r Package for the Guide 'Doing Meta-Analysis in r' (R Package Version 0.0.9000) Available online: https://dmetar.protectlab.org/ (accessed on 16 November 2023).
105. Wickham, H.; Seidel, D.; RStudio Scales: Scale Functions for Visualization 2022.
