# Supplementary material for: A Meta-Analysis of the Global Prevalence of Temporomandibular Disorders
Source: J Clin Med. 2024 Feb 28;13(5):1365. doi: 10.3390/jcm13051365 (PMC10931584; doi:10.3390/jcm13051365)

Figure S1. The results by forest plot prevalence of TMDs by continent.

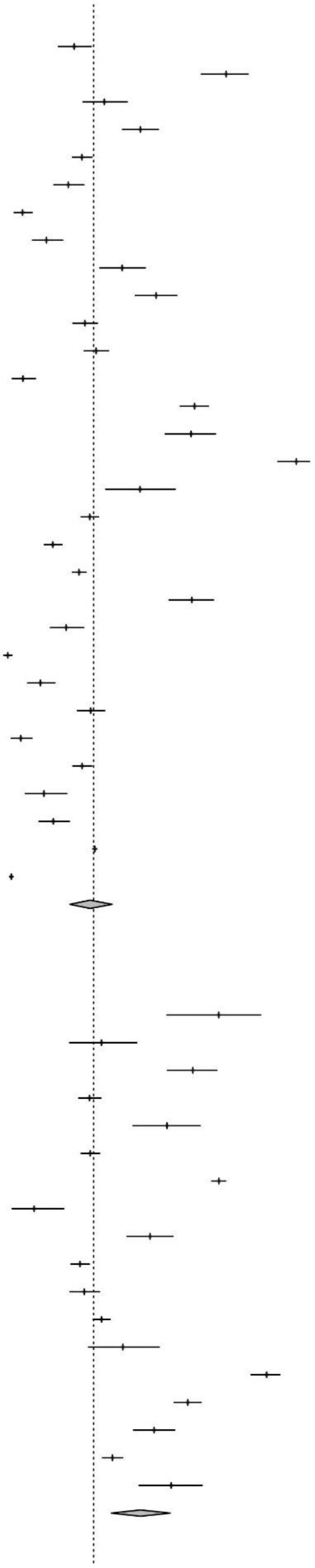

Study	Events	Total	Proportion	95%-CI
**Continent = Asia**				
Jomhawi J, 2021	114	396	0.29	[0.25; 0.33]
Bahrani F, 2012	140	200	0.70	[0.63; 0.76]
Srivastava K, 2021	91	246	0.37	[0.31; 0.43]
Habib S, 2015	187	400	0.47	[0.42; 0.52]
Marpaung C, 2018	365	1181	0.31	[0.28; 0.34]
Al-Khotani A, 2016	124	456	0.27	[0.23; 0.31]
Hongxing L, 2006	121	817	0.15	[0.13; 0.17]
Feteih R, 2006	82	385	0.21	[0.17; 0.26]
Natu V, 2018	102	244	0.42	[0.36; 0.48]
Taneja P, 2019	153	300	0.51	[0.45; 0.57]
Wu J, 2021	239	754	0.32	[0.28; 0.35]
Ebrahimi M, 2011	278	800	0.35	[0.32; 0.38]
Wu N, 2010	74	497	0.15	[0.12; 0.18]
Lei J, 2016	381	620	0.61	[0.58; 0.65]
Özdinç S , 2020	118	195	0.61	[0.53; 0.67]
Yasuda E, 2015	187	210	0.89	[0.84; 0.93]
Nourallah H, 1995	49	105	0.47	[0.37; 0.56]
Pow E, 1999	504	1526	0.33	[0.31; 0.35]
Verdonck A, 1994	272	1182	0.23	[0.21; 0.25]
Akhter R, 2008	715	2374	0.30	[0.28; 0.32]
Alketbi N, 2022	153	252	0.61	[0.55; 0.67]
Alrashdan M, 2019	98	368	0.27	[0.22; 0.31]
Talaat W, 2018	325	3009	0.11	[0.10; 0.12]
Kmeid E, 2020	87	443	0.20	[0.16; 0.24]
Yu Q, 2015	205	616	0.33	[0.30; 0.37]
Prakash J, 2022	86	600	0.14	[0.12; 0.17]
Nekora-Azak A, 2006	388	1253	0.31	[0.28; 0.34]
Alkhubaizi Q, 2022	41	199	0.21	[0.16; 0.27]
Karthik R, 2017	93	402	0.23	[0.19; 0.28]
Choi Y, 2002	9624	27978	0.34	[0.34; 0.35]
Song H, 2018	2065	17575	0.12	[0.11; 0.12]
**Random effects model**		**65583**	**0.33**	**[0.28; 0.39]**
Heterogeneity: $I^2 = 99\%$, $\tau^2 = 0.5297$, $p = 0$				
**Continent = South America**				
Pedroni C, 2003	34	50	0.68	[0.54; 0.79]
Fernandes Azevedo A, 2018	38	105	0.36	[0.28; 0.46]
Camacho J, 2014	122	200	0.61	[0.54; 0.68]
Bertoli F, 2018	308	934	0.33	[0.30; 0.36]
Medeiros R, 2020	61	113	0.54	[0.45; 0.63]
Júnior P, 2019	445	1342	0.33	[0.31; 0.36]
Oliveira A, 2006	1630	2396	0.68	[0.66; 0.70]
Stefano A, 2022	21	117	0.18	[0.12; 0.26]
Figueiredo Ribeiro D, 2020	121	245	0.49	[0.43; 0.56]
Franco-Micheloni A, 2015	397	1307	0.30	[0.28; 0.33]
Khan K, 2020	165	523	0.32	[0.28; 0.36]
Progiante P, 2015	595	1643	0.36	[0.34; 0.39]
Mello V, 2014	42	100	0.42	[0.33; 0.52]
Fonseca F, 2022	311	384	0.81	[0.77; 0.85]
Campos J, 2014	418	701	0.60	[0.56; 0.63]
Sampaio N, 2017	155	307	0.50	[0.45; 0.56]
Gonçalves D, 2010	482	1230	0.39	[0.36; 0.42]
Dallanora A, 2012	70	127	0.55	[0.46; 0.64]
**Random effects model**		**11824**	**0.47**	**[0.39; 0.55]**
Heterogeneity: $I^2 = 98\%$, $\tau^2 = 0.4681$, $p < 0.01$				

**Continent = North America**			
Ramírez-Caro S, 2015	31	150	0.21 [0.15; 0.28]
Khan K, 2020	73	312	0.23 [0.19; 0.28]
Mendiburu-Zavala C, 2020	156	210	0.74 [0.68; 0.80]
Isong U, 2008	1345	30978	0.04 [0.04; 0.05]
Moyaho-Bernal A, 2010	78	235	0.33 [0.27; 0.39]
**Random effects model**		**31885**	**0.26 [0.06; 0.65]**
Heterogeneity: $I^2 = 100\%$, $\tau^2 = 3.5415$, $p < 0.01$			
**Continent = Europe**			
Khan K, 2020	190	597	0.32 [0.28; 0.36]
Graue A, 2016	20	167	0.12 [0.08; 0.18]
Qvintus V, 2020	546	1577	0.35 [0.32; 0.37]
Perrotta S, 2019	103	700	0.15 [0.12; 0.18]
Hongxing L, 2016	42	817	0.05 [0.04; 0.07]
Paduano S, 2020	99	361	0.27 [0.23; 0.32]
Tecco S, 2011	278	1134	0.25 [0.22; 0.27]
Tecco S, 2019	250	567	0.44 [0.40; 0.48]
Marpaung C, 2018	851	3940	0.22 [0.20; 0.23]
Wu N, 2010	73	561	0.13 [0.10; 0.16]
Stefano A, 2022	40	138	0.29 [0.22; 0.37]
Loster J, 2017	69	260	0.27 [0.22; 0.32]
Nilsson I, 2005	1214	28899	0.04 [0.04; 0.04]
Wieckiewicz M, 2020	104	213	0.49 [0.42; 0.56]
Vainionpää R, 2019	84	100	0.84 [0.75; 0.90]
Storm C, 2006	156	487	0.32 [0.28; 0.36]
Ciancaglini R, 2001	266	483	0.55 [0.51; 0.59]
Jussila P, 2017	671	1962	0.34 [0.32; 0.36]
Carlsson G, 2014	1025	8619	0.12 [0.11; 0.13]
Yekkalam N, 2014	397	600	0.66 [0.62; 0.70]
Yekkalam N, 2014	370	600	0.62 [0.58; 0.65]
Hadler-Olsen E, 2021	690	1946	0.35 [0.33; 0.38]
Gesch D, 2004	2140	4289	0.50 [0.48; 0.51]
Rantala M, 2003	362	1339	0.27 [0.25; 0.29]
Banafa A, 2020	117	1210	0.10 [0.08; 0.11]
Barbosa C, 2021	543	1381	0.39 [0.37; 0.42]
**Random effects model**		**62947**	**0.29 [0.21; 0.39]**
Heterogeneity: $I^2 = 100\%$, $\tau^2 = 1.3513$, $p = 0$			
**Random effects model**		**172239**	**0.34 [0.29; 0.39]**
Heterogeneity: $I^2 = 100\%$, $\tau^2 = 1.0579$, $p = 0$			
Test for subgroup differences: $\chi^2_3 = 10.54$, df = 3, ($p = 0.01$)			

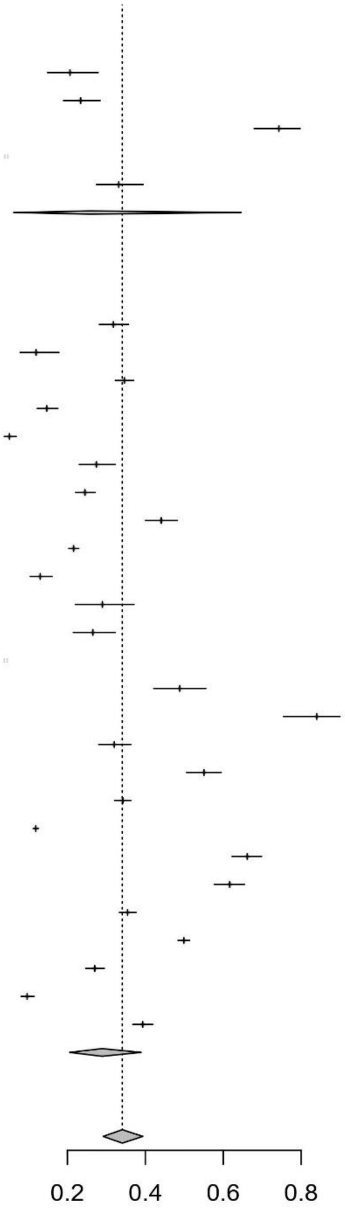

Figure S2. The results by forest plot prevalence of TMDs by age.

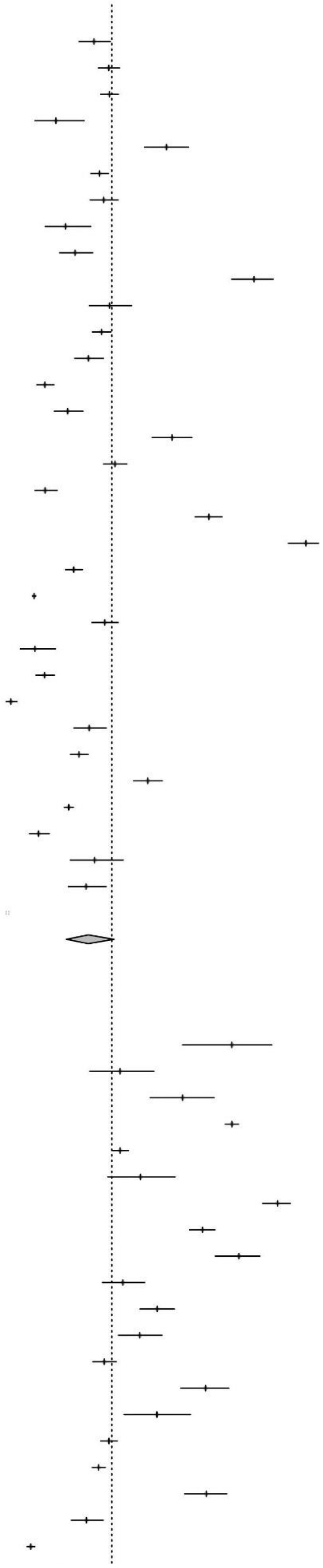

Study	Events	Total	Proportion	95%-CI
**Age = up to 18 yrs.**				
Jomhawi J, 2021	114	396	0.29	[0.25; 0.33]
Bertoli F, 2018	308	934	0.33	[0.30; 0.36]
Júnior P, 2019	445	1342	0.33	[0.31; 0.36]
De Stefano A, 2022	21	117	0.18	[0.12; 0.26]
Figueiredo Ribeiro D, 2020	121	245	0.49	[0.43; 0.56]
Franco-Micheloni A, 2015	397	1307	0.30	[0.28; 0.33]
Khan K, 2020	165	523	0.32	[0.28; 0.36]
Ramírez-Caro S, 2015	31	150	0.21	[0.15; 0.28]
Khan K, 2019	73	312	0.23	[0.19; 0.28]
Mendiburu-Zavala C, 2020	156	210	0.74	[0.68; 0.80]
Moyaho-Bernal A, 2010	78	235	0.33	[0.27; 0.39]
Marpaung C, 2018	365	1181	0.31	[0.28; 0.34]
Al-Khotani A, 2016	124	456	0.27	[0.23; 0.31]
Hongxing L, 2016	121	817	0.15	[0.13; 0.17]
Feteih R, 2006	82	385	0.21	[0.17; 0.26]
Taneja P, 2019	153	300	0.51	[0.45; 0.57]
Ebrahimi M, 2011	278	800	0.35	[0.32; 0.38]
Wu N, 2010	74	497	0.15	[0.12; 0.18]
Lei J, 2016	381	620	0.61	[0.58; 0.65]
Yasuda E, 2015	187	210	0.89	[0.84; 0.93]
Verdonck A, 1994	272	1182	0.23	[0.21; 0.25]
Song H, 2018	2065	17575	0.12	[0.11; 0.12]
Khan K, 2019	190	597	0.32	[0.28; 0.36]
Graue A, 2016	20	167	0.12	[0.08; 0.18]
Perrotta S, 2019	103	700	0.15	[0.12; 0.18]
Hongxing L, 2016	42	817	0.05	[0.04; 0.07]
Paduano S, 2020	99	361	0.27	[0.23; 0.32]
Tecco S, 2011	278	1134	0.25	[0.22; 0.27]
Tecco S, 2019	250	567	0.44	[0.40; 0.48]
Marpaung C, 2018	851	3940	0.22	[0.20; 0.23]
Wu N, 2010	73	561	0.13	[0.10; 0.16]
De Stefano A, 2022	40	138	0.29	[0.22; 0.37]
Loster J, 2017	69	260	0.27	[0.22; 0.32]
Nilsson I, 2005	1214	28899	0.04	[0.04; 0.04]
**Random effects model**		**67935**	**0.27**	**[0.21; 0.34]**
Heterogeneity: $I^2 = 99\%$, $\tau^2 = 1.0283$, $p = 0$				
**Age = 18-60 yrs.**				
Pedroni C, 2003	34	50	0.68	[0.54; 0.79]
Fernandes Azevedo A, 2018	38	105	0.36	[0.28; 0.46]
Medeiros R, 2020	61	113	0.54	[0.45; 0.63]
Oliveira A, 2006	1630	2396	0.68	[0.66; 0.70]
Progiante P, 2015	595	1643	0.36	[0.34; 0.39]
Mello V, 2014	42	100	0.42	[0.33; 0.52]
Fonseca F, 2022*	311	384	0.81	[0.77; 0.85]
Campos J, 2014	418	701	0.60	[0.56; 0.63]
Bahrani F, 2012	140	200	0.70	[0.63; 0.76]
Srivastava K, 2021	91	246	0.37	[0.31; 0.43]
Habib S, 2015	187	400	0.47	[0.42; 0.52]
Natu V, 2018	102	244	0.42	[0.36; 0.48]
Wu J, 2021	239	754	0.32	[0.28; 0.35]
Özdinç S , 2020	118	195	0.61	[0.53; 0.67]
Nourallah H, 1995	49	105	0.47	[0.37; 0.56]
Pow E, 2001	504	1526	0.33	[0.31; 0.35]
Akhter R, 2008	715	2374	0.30	[0.28; 0.32]
Alketbi N, 2022	153	252	0.61	[0.55; 0.67]
Alrashdan M, 2019	98	368	0.27	[0.22; 0.31]
Talaat W, 2018	325	3009	0.11	[0.10; 0.12]

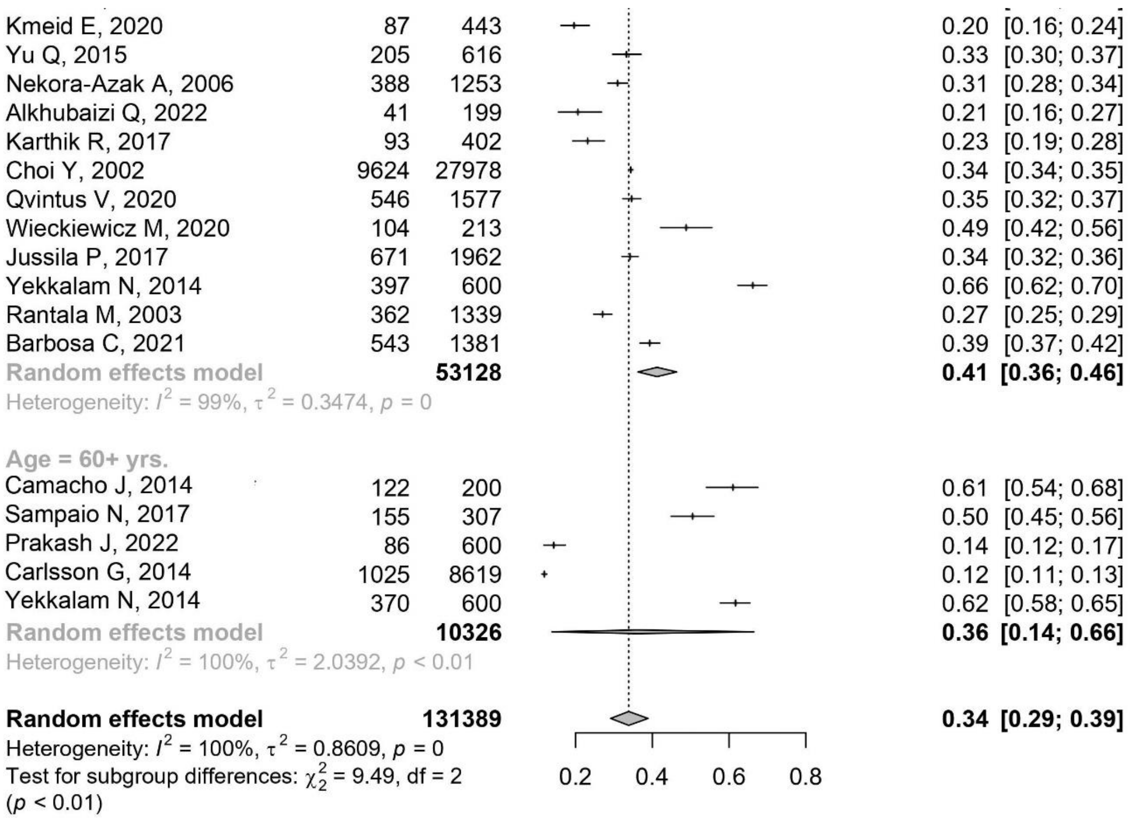
Kmeid E, 2020 87 443 0.20 [0.16; 0.24]
Yu Q, 2015 205 616 0.33 [0.30; 0.37]
Nekora-Azak A, 2006 388 1253 0.31 [0.28; 0.34]
Alkhubaizi Q, 2022 41 199 0.21 [0.16; 0.27]
Karthik R, 2017 93 402 0.23 [0.19; 0.28]
Choi Y, 2002 9624 27978 0.34 [0.34; 0.35]
Qvintus V, 2020 546 1577 0.35 [0.32; 0.37]
Wieckiewicz M, 2020 104 213 0.49 [0.42; 0.56]
Jussila P, 2017 671 1962 0.34 [0.32; 0.36]
Yekkalam N, 2014 397 600 0.66 [0.62; 0.70]
Rantala M, 2003 362 1339 0.27 [0.25; 0.29]
Barbosa C, 2021 543 1381 0.39 [0.37; 0.42]
Random effects model 53128 0.41 [0.36; 0.46]
Heterogeneity: I^2 = 99%, τ^2 = 0.3474, p = 0
Age = 60+ yrs.
Camacho J, 2014 122 200 0.61 [0.54; 0.68]
Sampaio N, 2017 155 307 0.50 [0.45; 0.56]
Prakash J, 2022 86 600 0.14 [0.12; 0.17]
Carlsson G, 2014 1025 8619 0.12 [0.11; 0.13]
Yekkalam N, 2014 370 600 0.62 [0.58; 0.65]
Random effects model 10326 0.36 [0.14; 0.66]
Heterogeneity: I^2 = 100%, τ^2 = 2.0392, p < 0.01
Random effects model 131389 0.34 [0.29; 0.39]
Heterogeneity: I^2 = 100%, τ^2 = 0.8609, p = 0
Test for subgroup differences: χ^2_2 = 9.49, df = 2
(p < 0.01)
0.2 0.4 0.6 0.8

Figure S3. The results by forest plot prevalence of TMDs continent (Asia) and age.

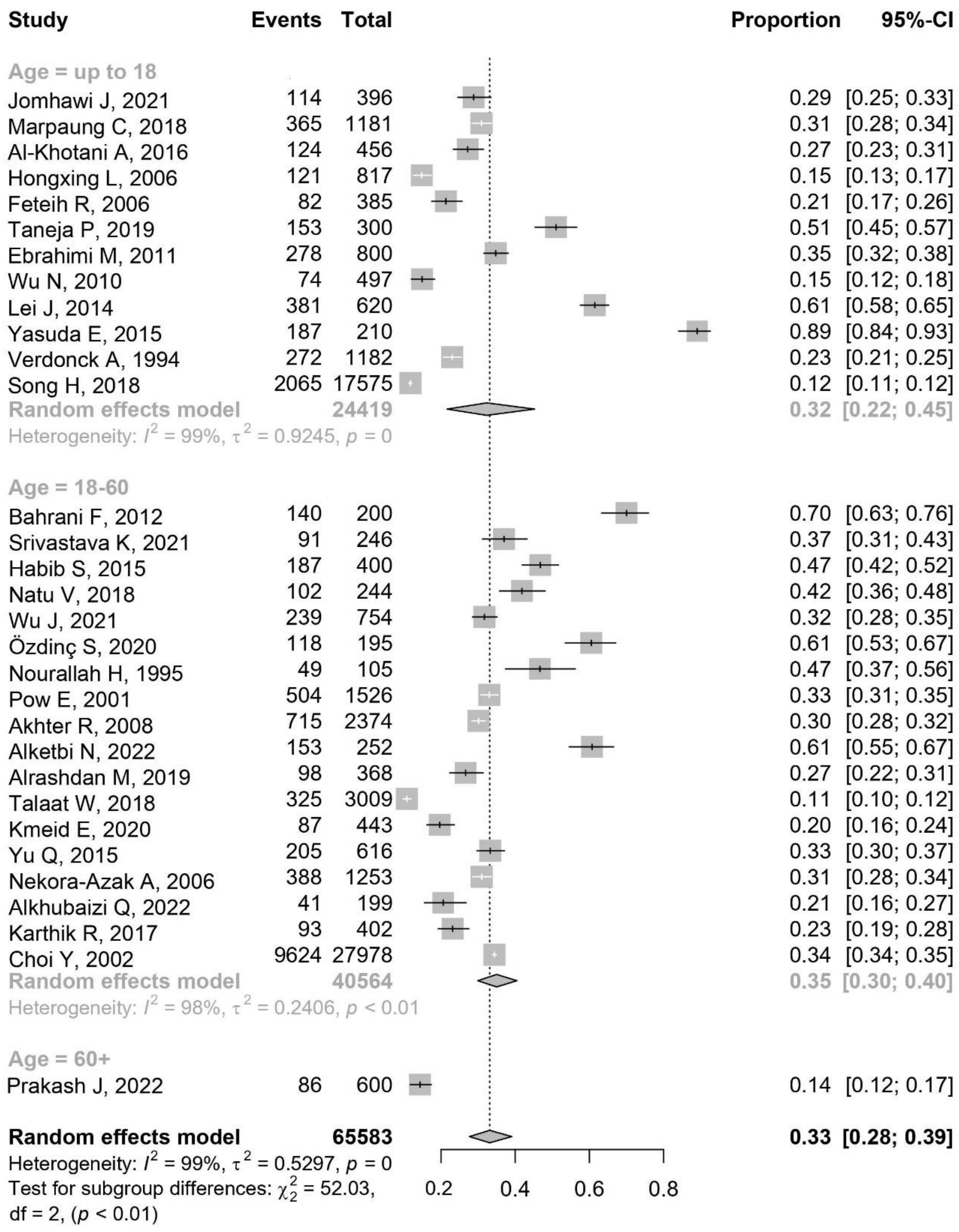

Figure S4. The results by forest plot prevalence of TMDs continent (South America) and age.

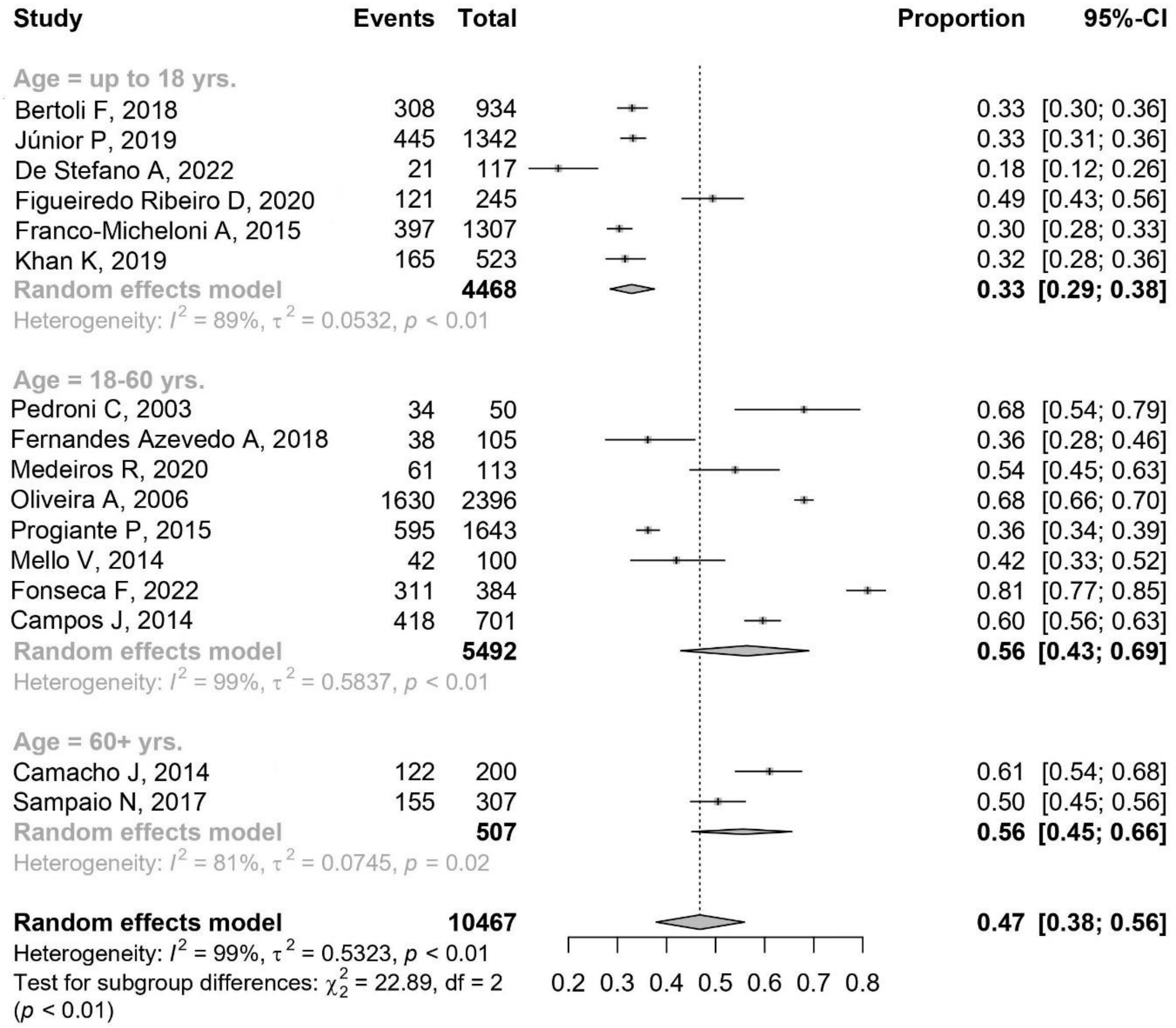

Figure S5. The results by forest plot prevalence of TMDs continent (North America) and age.

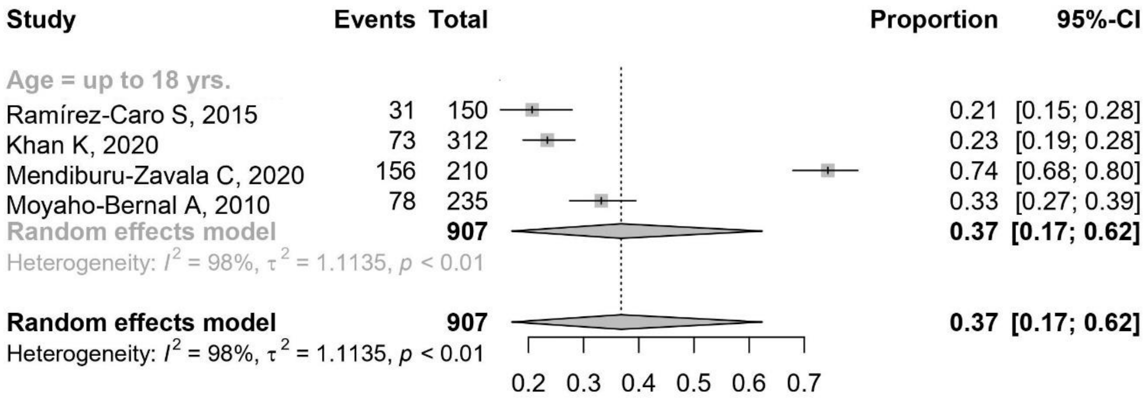

Figure S6. The results by forest plot prevalence of TMDs continent (Europe) and age.

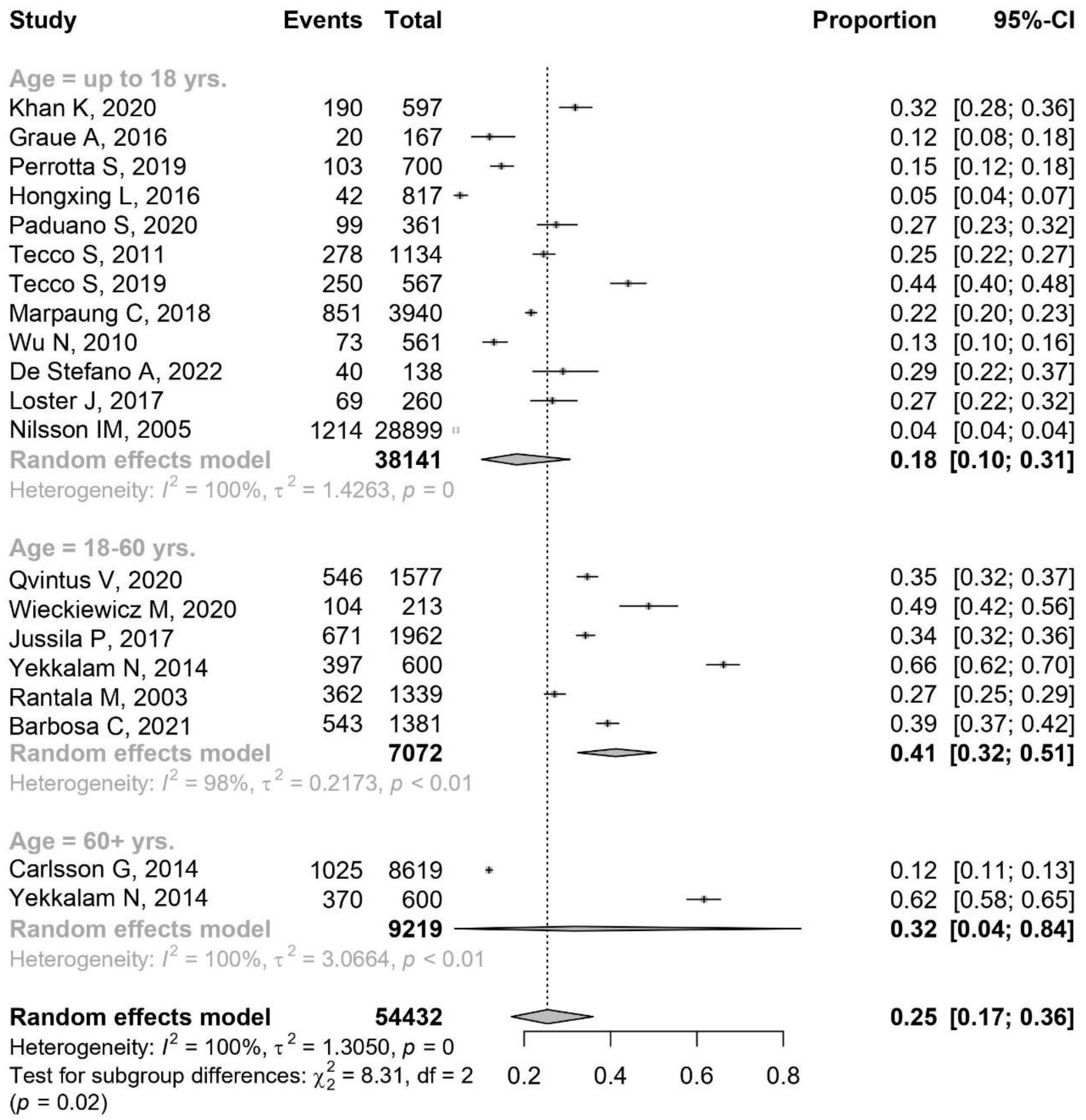

Supplement: Supplementary file 1 [file jcm-13-01365-s001.zip › Supplementary Material S4.pdf]
