# Supplementary material for: A Meta-Analysis of the Global Prevalence of Temporomandibular Disorders
Source: J Clin Med. 2024 Feb 28;13(5):1365. doi: 10.3390/jcm13051365 (PMC10931584; doi:10.3390/jcm13051365)
Supplement: Supplementary file 1 [file jcm-13-01365-s001.zip › Supplementary Material S5.pdf]

Table S1. Rejected studies from the meta-analysis.

Author	Year	Continent	n	Reason for rejection	
Adegbiji et al.	2021	Africa	65	Not enough research	[1]
Rubin et al.	2018	Africa	153		[2]
Lung, J. et al.	2018	Australia	392		[3]
Zwiri and Al-Omiri	2016	Asia	489	Influential case	[4]

References

1. Adegbiji, W.A.; Olajide, G.T.; Agbesanwa, A.T.; Banjo, O.O. Otological Manifestation of Temporomandibular Joint Disorder in Ekiti, a Sub-Saharan African Country. *J. Int. Med. Res.* **2021**, *49*, 300060521996517, doi:10.1177/0300060521996517.
2. Friedman Rubin, P.; Erez, A.; Peretz, B.; Birenboim-Wilensky, R.; Winocur, E. Prevalence of Bruxism and Temporomandibular Disorders among Orphans in Southeast Uganda: A Gender and Age Comparison. *CRANIO®* **2018**, *36*, 243–249, doi:10.1080/08869634.2017.1331784.
3. Lung, J.; Bell, L.; Heslop, M.; Cuming, S.; Ariyawardana, A. Prevalence of Temporomandibular Disorders among a Cohort of University Undergraduates in Australia. *J. Investig. Clin. Dent.* **2018**, *9*, e12341, doi:10.1111/jicd.12341.
4. Zwiri, A.M.A.; Al-Omiri, M.K. Prevalence of Temporomandibular Joint Disorder among North Saudi University Students. *CRANIO®* **2016**, *34*, 176–181, doi:10.1179/2151090315Y.0000000007.
